# Additive contributions of melanopsin and both cone types provide broadband sensitivity to mouse pupil control

**DOI:** 10.1186/s12915-018-0552-1

**Published:** 2018-07-31

**Authors:** Edward A. Hayter, Timothy M. Brown

**Affiliations:** Faculty of Biology, Medicine and Health, University of Manchester, AV Hill Building, Oxford Road, Manchester, M13 9PT UK

**Keywords:** Melanopsin, Pupil, Photoreceptor, Colour vision, Mouse, Electrophysiology, Silent substitution, Olivary pretectal nucleus

## Abstract

**Background:**

Intrinsically photosensitive retinal ganglion cells (ipRGCs) drive an array of non-image-forming (NIF) visual responses including circadian photoentrainment and the pupil light reflex. ipRGCs integrate extrinsic (rod/cone) and intrinsic (melanopsin) photoreceptive signals, but the contribution of cones to ipRGC-dependent responses remains incompletely understood. Given recent data revealing that cone-derived colour signals influence mouse circadian timing and pupil responses in humans, here we set out to investigate the role of colour information in pupil control in mice.

**Results:**

We first recorded electrophysiological activity from the pretectal olivary nucleus (PON) of anaesthetised mice with a red-shifted cone population (*Opn1mw*^*R*^) and mice lacking functional cones (*Cnga3*^*−/−*^) or melanopsin (*Opn1mw*^*R*^*; Opn4*^*−/−*^). Using multispectral stimuli to selectively modulate the activity of individual opsin classes, we show that PON cells which receive ipRGC input also exhibit robust S- and/or L-cone opsin-driven activity. This population includes many cells where the two cone opsins drive opponent responses (most commonly excitatory/ON responses to S-opsin stimulation and inhibitory/OFF responses to L-opsin stimulation). These cone inputs reliably tracked even slow (0.025 Hz) changes in illuminance/colour under photopic conditions with melanopsin contributions becoming increasingly dominant for higher-contrast/lower temporal frequency stimuli. We also evaluated consensual pupil responses in awake animals and show that, surprisingly, this aspect of physiology is insensitive to chromatic signals originating with cones. Instead, by contrast with the situation in humans, signals from melanopsin and both cone opsins combine in a purely additive manner to drive pupil constriction in mice.

**Conclusion:**

Our data reveal a key difference in the sensory control of the mouse pupil relative to another major target of ipRGCs—the circadian clock. Whereas the latter uses colour information to help estimate time of day, the mouse pupil instead sums signals across cone opsin classes to provide broadband spectral sensitivity to changes in illumination. As such, while the widespread co-occurrence of chromatic responses and melanopsin input in the PON supports a close association between colour discrimination mechanisms and NIF visual processing, our data suggest that colour opponent PON cells in the mouse contribute to functions other than pupil control.

**Electronic supplementary material:**

The online version of this article (10.1186/s12915-018-0552-1) contains supplementary material, which is available to authorized users.

## Background

In addition to supporting visual perception, output from the mammalian retina drives an array of subconscious physiological and behavioural modifications including regulation of internal circadian clocks, hormone secretion and pupillomotor responses [1]. Given their fundamental role in shaping animal physiology and their clinical relevance [2], defining the sensory signals that drive such ‘non-image-forming’ (NIF) visual responses has been a long-standing goal of sensory biology [3]. A key advance in this regard was the realisation that such responses rely on signals from a specialised subset of intrinsically photosensitive retinal ganglion cells (ipRGCs) that innervate key retinorecipient nuclei [4, 5] including the suprachiasmatic nuclei (SCN; site of master circadian clock) and the pretectal olivary nuclei (PON; central relay controlling pupillary responses). Importantly, however, ipRGCs also receive visual information from rods and cones [6–8] and specific contributions of these outer retinal signals relative to the intrinsic, melanopsin-derived, light response remain incompletely understood [9].

In particular, the contribution of cones to ipRGC-dependent physiological responses has proved hard to define. On the one hand, there is abundant evidence for cone-derived responses in ipRGCs and the brain regions they innervate [10–17]. However, from a conceptual standpoint, the rapid adaptation that characterises cone photoreception [18] reduces the extent to which cones can provide accurate information about ambient light levels (which are assumed to be especially important for ipRGC-driven responses). Accordingly, while cone photoreceptors do display some degree of irradiance-dependent maintained responses that could provide information about steady-state light intensity [19, 20], investigations of ipRGC-dependent physiological/behavioural responses in mice have often failed to find evidence for a major contribution from cones [21–25]. Until recently then, a prevailing view has been that ipRGCs primarily use a combination of rods and melanopsin to track changes in ambient light intensity [26], with cone contributions limited to tracking more rapid fluctuations in illumination (visual contrast).

As part of on-going work aimed at better understanding how cone photoreception contributes to NIF visual responses, we recently demonstrated that cones in fact contribute a quite distinct sensory dimension to mouse circadian photoreception; providing information about daily changes in the spectral composition (colour) of daylight [27]. At the cellular level, this is reflected by a sizeable population of SCN neurons that exhibit antagonistic responses to stimulation of the two classes of mouse cone opsin, i.e. colour-opponent behaviour. Consistent with those findings, a subtype of ipRGC exhibiting cone-dependent colour-opponency was recently identified in the mouse retina [28]. Alongside previous work revealing colour-opponency also in primate ipRGCs [29] and human pupillary responses [30–32], it now appears that an especially important contribution of cone input to the NIF visual system may in fact be to provide information about colour [33]. Nonetheless, to date there have been comparatively few explicit investigations of how colour influences NIF visual responses in any mammalian species. Accordingly, here we set out to determine whether the chromatic influences we identified for the mouse circadian system [27] extend also to other ipRGC-dependent responses in this species. To this end, here we employ carefully calibrated visual stimuli that allow for selective manipulation of individual photoreceptor classes (‘silent substitution’) alongside a well-validated mouse model (*Opn1mw*^*R*^) with long-wavelength-shifted cone spectral sensitivity [24, 34] to comprehensively define the influence of cones on a major target of ipRGC regulation—neurophysiological activity in the PON and the mouse pupil.

## Results

### Identification of melanopsin-responsive pretectal neurons

**Fig. 1:**
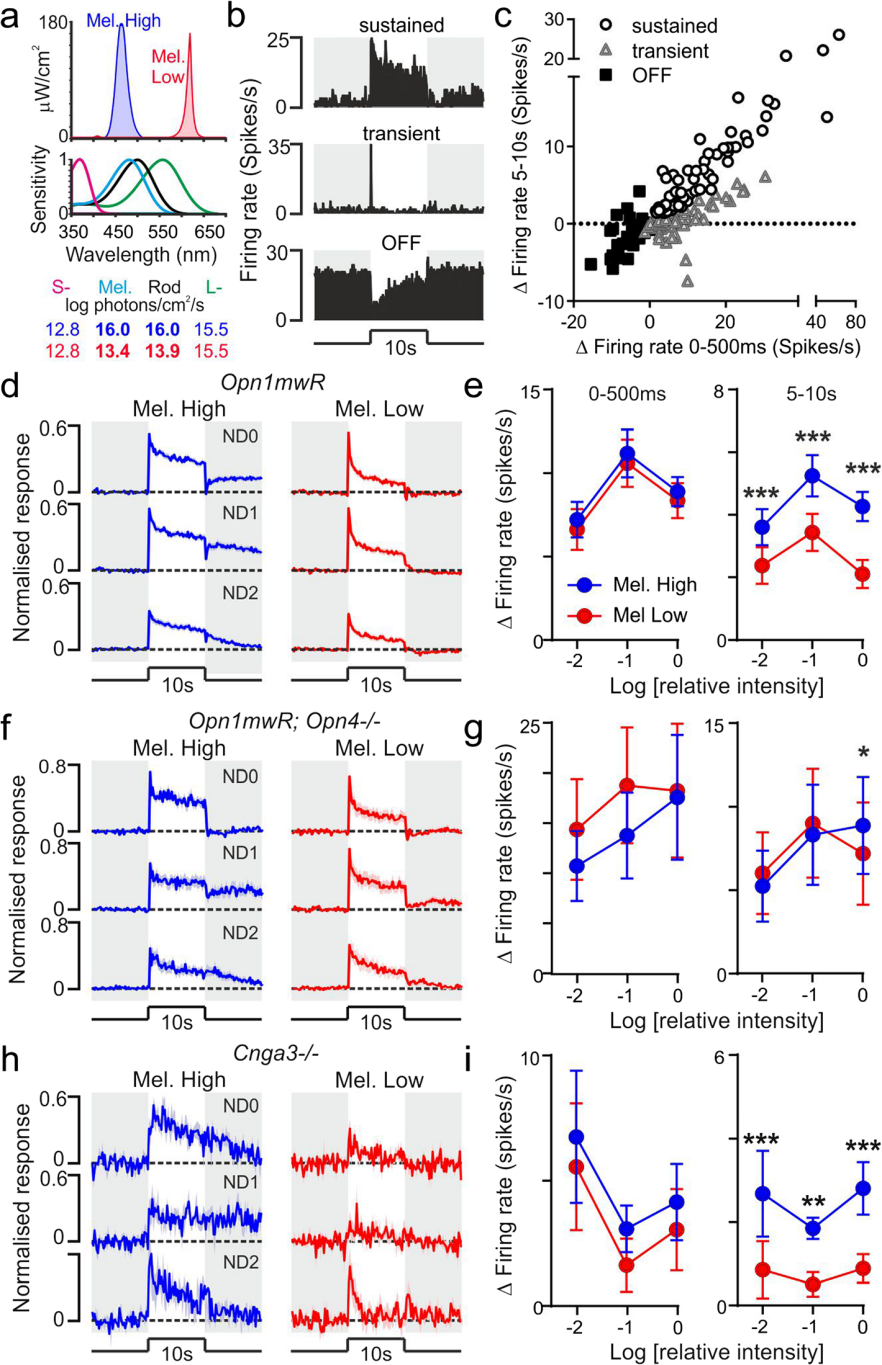
Identification of melanopsin-responsive pretectal neurons. **a** Spectral power distributions for cone-matched stimuli designed to strongly (Mel. High) or more weakly (Mel. Low) activate melanopsin. Lower panel shows opsin sensitivity in *Opn1mw*^*R*^ mice, corrected for prereceptoral filtering, and the log effective photon flux calculated for each opsin. **b** Example of responses of ‘sustained’, ‘transient’ and ‘OFF’ cells to Mel. High stimuli presented at ND1 (1 log unit reduction relative to **a**). **c** Scatter plot showing maximal change in firing evoked by Mel. High stimuli during first 500 ms and last 5 s of a 10-s light step for visually responsive cells identified in *Opn1mw*^*R*^ mice, classified according to response type (sustained, *n* = 72, transient, *n* = 121, OFF, *n* = 37). **d**, **f**, **h** Mean ± SEM normalised change in firing for Mel High and Low steps across three logarithmically spaced intensities for melanopsin-responsive sustained cell populations in *Opn1mw*^*R*^ (**d**; *n* = 60), *Cnga3*^*−/−*^ (**h**; *n* = 5) and their equivalents in *Opn1mw*^*R*^*; Opn4*^*−/−*^ (**f,** *n* = 9). Shaded regions represent epochs of darkness. **e**, **g**, **i** Mean ± SEM change in firing observed during early (0–500 ms) and late components (last 5 s) of the Mel. High and Low light steps for corresponding cell populations in **d, f** and **h**. Data were analysed by two-way RM ANOVA with Sidak’s post-tests at each intensity when significant main effects of stimulus or StimulusxIrradiance were identified. *,** and *** = *P* < 0.05, 0.01 and 0.001 respectively, otherwise *P* > 0.05

We first set out to identify neurons in the mouse pretectum that received input from ipRGCs. To this end, we performed multielectrode (32 channel) recordings from the PON and surrounding pretectum of 26 anaesthetised *Opn1mw*^*R*^ mice. Although the pretectum receives input from both ipRGCs and other RGC types [35, 36], a characteristic feature of melanopsin phototransduction is a sluggish and sustained elevation in firing in response to high intensity short-wavelength (‘blue’) light [5]. Accordingly, to screen for cells likely to receive input from ipRGCs, we first evaluated responses to monochromatic 460-nm light steps (10 s duration from darkness) across a range of intensities (Fig. 1a; 14–16 Log melanopsin effective photons/cm^2^/s; termed here ‘Mel High’) predicted to robustly activate melanopsin-based responses in all known classes of ipRGCs [37–39].

Across all primary retinorecipient targets, visual responses driven by rods and cones are characterised by an acute increase in firing rate within a few hundred milliseconds following stimulus onset that typically decays substantially on prolonged stimulation [11, 12, 16, 17, 27, 40]. By contrast, where present, response elements originating with melanopsin build up over several seconds and persist even under very long exposure [7, 11, 16, 17, 40]. We first then quantified early and later components of the response to bright 460-nm light steps (0–500 ms and 5–10 s following stimulus onset respectively) to identify neurons with the sustained changes in firing consistent with melanopsin input (Fig. 1b).

As expected, based on previous data [11], a substantial number of the pretectal neurons we isolated from these multielectrode recordings (*n* = 72/230 visually responsive cells) exhibited sustained increases in firing in response to such stimuli (Fig. 1b, c). By contrast, the remaining cells exhibited responses incompatible with a significant contribution from ipRGCs (Fig. 1b, c): the majority (*n* = 121/158) exhibited only very transient increases in firing at the onset and offset of the light step (as reported previously; [11]), while a smaller subset (*n* = 37) exhibited light-driven decreases in firing rate (OFF responses).

To more specifically establish that the population of cells exhibiting sustained responses to Mel High stimuli did indeed correspond to those receiving input from ipRGCs, we next evaluated their responses to polychromatic light steps, matched to provide identical stimulation of L- and S-cones but a significantly weaker impact on melanopsin (Fig. 1a; ~ 500-fold weaker, termed here ‘Mel Low’). We chose this approach, initially, since we predicted it would allow us to evaluate the impact of much larger variations in melanopsin excitation than are achievable using more selective melanopsin-isolating stimuli, without encountering the rod-intrusion that can occur following light-adaptation [16, 40, 41]. Hence, while the Mel Low/High stimuli used here also differed substantially in their impact on rods, our expectation was that, at the two higher intensities tested, both should be sufficiently bright to produce a saturating rod response when presented as light steps from darkness (≥ 12.9 and 15 Log rod effective photons/cm^2^/s for Mel Low and High respectively; c.f. [42]).

Consistent with our expectation that Mel High and Low stimuli should produce equivalent rod/cone-mediated responses, light-responsive cells whose properties were incompatible with ipRGC input (those with transient or OFF responses) exhibited equivalent responses to both stimuli (Additional file 1: Figure S1a,b). Similarly, as expected given the very sluggish nature of melanopsin phototransduction, for the majority of the cells with sustained responses (*n* = 60/72), the initial increase in firing (peak occurring during first 500 ms of light step) was statistically equivalent for Mel Low/High at one or both of the top two intensities tested (within-cell *t* tests across 10 trials at each stimulus). Further analysis of this group of cells indicated that, in fact, there was no detectable effect of stimulus on early response components (mean firing during first 500 ms) at any intensity (Fig. 1d, e; two-way RM ANOVA with Sidak’s post-tests, all *P* > 0.05). Importantly, however, Mel High/Low responses diverged at later timescales (Fig. 1d) such that, over the last 5 s of the response, the Mel High-evoked increase in firing was significantly greater at all intensities tested (Fig. 1e; two-way RM ANOVA with Sidak’s post-tests). Based on this pronounced and selective enhancement of late components of the Mel High response (across a range where melanopsin phototransduction is active; [37]), we consider this group of cells melanopsin responsive (MR). These observations align well with the kinetics of PON neuronal responses observed previously in rodless/coneless mice [11]. This conclusion is also further supported by additional data reported below and later in the manuscript (Figs. 5 and 7 and associated additional files), including the anatomical location of such cells which, as expected based on known ipRGC projections [43, 44], were strongly clustered in the region of the PON.

By contrast to the above, we also observed a small number of cells (*n* = 12) with sustained responses where both early and later components of the responses were significantly enhanced for Mel High stimuli, an effect that was most apparent at the highest intensity tested (Additional file 2: Figure S2a,b). Although we cannot definitively rule out a melanopsin contribution to the responses of this group of cells, this difference even in the earliest portions of the response suggests contributions from a photoreceptor other than melanopsin (presumably an unexpected contribution from rods; see below). Accordingly, for subsequent analysis, we consider this rare group of cells (~ 5% of the light-responsive cells identified here) as non-MR.

To provide further confidence in our classification of cells as melanopsin responsive, we also performed parallel sets of experiments in melanopsin knockout red cone animals (*Opn1mw*^*R*^*; Opn4*^*−/−*^; *n* = 5 mice) and mice lacking functional cone photoreception (*Cnga3*^*−/−*^; *n* = 4). Among both groups of mice, a small population of cells (*n* = 8/41 and *n* = 3/24 for *Opn1mw*^*R*^*; Opn4*^*−/−*^ and *Cnga3*^*−/−*^ respectively) exhibited behaviour equivalent to that described above: a global enhancement in responses to the Mel High vs. Low stimulus that emerged at higher intensities (Additional file 2: Figure S2c-f). Since we observe this behaviour in recordings from animals lacking either cone or melanopsin phototransduction, we conclude this must reflect an unexpected difference in rod-mediated responses that appears in certain cells under high light intensities. This may reflect the emergence of bleaching adaptation [41] or perhaps even the contribution of an atypical phototransduction pathway [45]. In either case, the impact of such a mechanism appears to be sufficiently restricted (being essentially absent from the other classes of visually responsive cells we recorded; Fig. 1f–i; Additional file 1: Figure S1) that it does not significantly interfere with our ability to identify MR cells.

Of particular note here then are the more commonly encountered sustained cells exhibiting statistically identical initial increases in firing to Mel High/Low steps at high stimulus intensities (*n* = 9 and *n* = 5 for *Opn1mw*^*R*^*; Opn4*^*−/−*^ and *Cnga3*^*−/−*^ respectively). As for the large population of *Opn1mw*^*R*^ cells matching these criteria (i.e. MR cells), early components of the responses of both *Opn1mw*^*R*^*; Opn4*^*−/−*^ (Fig. 1f, g) and *Cnga3*^*−/−*^ cells (Fig. 1h, i) to Mel High vs. Low stimuli were in fact equivalent at all intensities (two-way RM ANOVAs with Sidak’s post-tests, all *P* > 0.05). Cells in *Cnga3*^*−/−*^ animals (where melanopsin remains functional) also retained the pronounced enhancement across later components of the Mel High response at all intensities (Fig. 1h, i; two-way RM ANOVA with Sidak’s post-tests). By contrast, responses of *Opn1mw*^*R*^*; Opn4*^*−/−*^ cells were qualitatively different (Fig. 1f, g), instead showing statistically equivalent responses at all but the highest intensity where Mel Low responses were very marginally reduced (Sidak’s post-test, *P* = 0.03; presumably reflecting the threshold appearance of a mechanism equivalent to that illustrated in Additional file 2: Figure S2). Collectively then, these data support our classification of cells as MR and provide further confidence that elements of the *Opn1mw*^*R*^ MR-cell responses ascribed to melanopsin do not instead reflect significant stimulus-related difference in rod/cone activation.

### Cone influences on melanopsin-responsive pretectal neurons

**Fig. 2:**
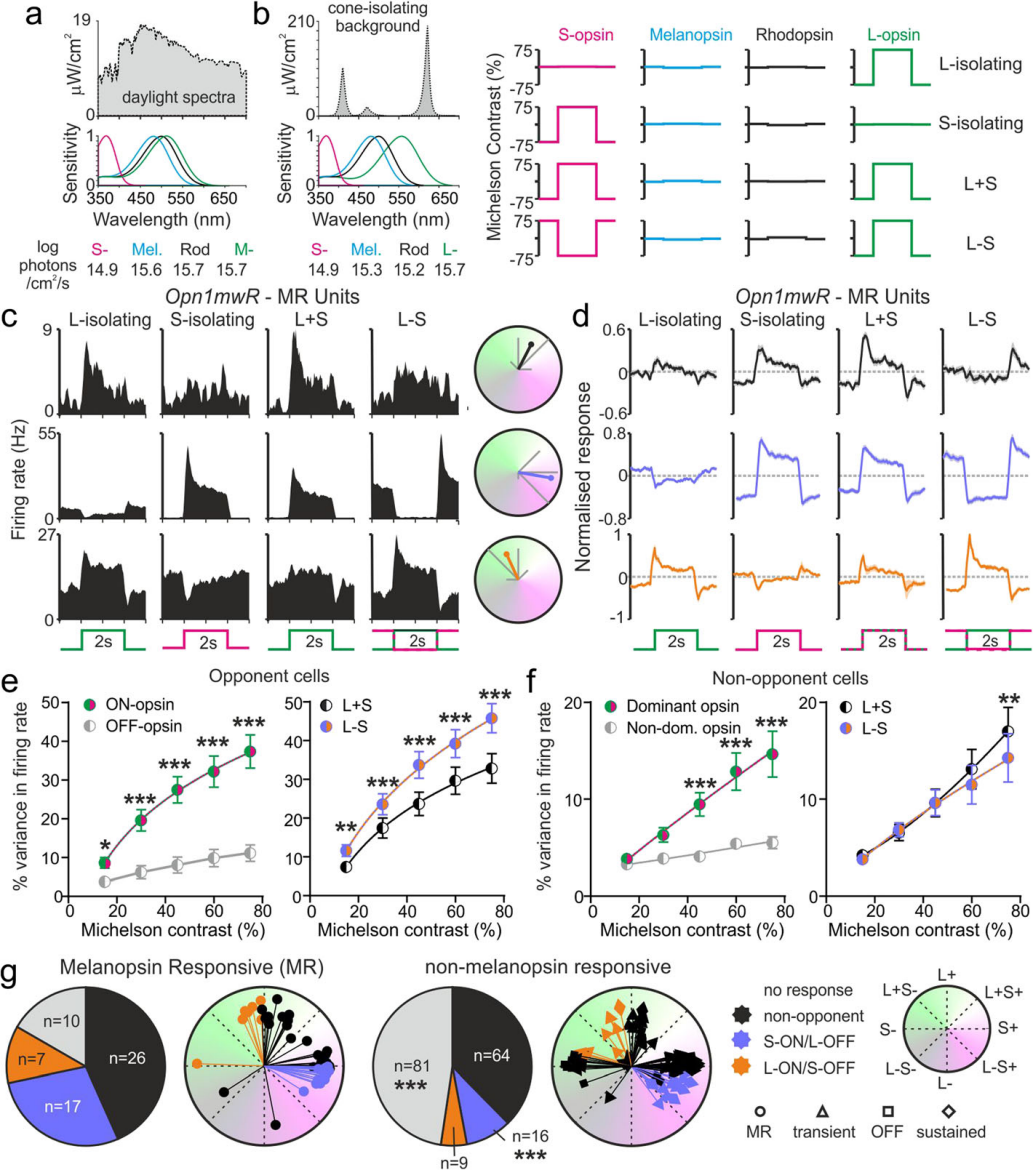
Opponent cone input is common among melanopsin-responsive pretectal neurons. **a** Spectral power distribution of daylight (7–8° solar elevation on clear days, from [27]) and effective photon flux for wildtype mouse opsins. **b** Spectra for the background stimulus used to generate cone-isolating stimuli, corresponding effective photon flux for *Opn1mw*^*R*^ mouse opsins and maximal contrasts for each produced by cone-isolating stimuli (see methods for additional details). **c** Left: Example responses of MR units to 75% contrast cone-isolating stimuli (30 trials) illustrating non-opponent (top), S-ON/L-OFF (middle) and L-ON/S-OFF (bottom) behaviour. Right: opsin preference plots showing normalised response across each stimulus-space dimension (grey lines; see ‘Methods’) and resulting mean vector, with vertical and horizontal axes corresponding to pure L- and S-opsin-driven responses and diagonals reflecting equally weighted opponent or non-opponent contributions (as indicated by key in left part of **g**). **d** Mean ± SEM baseline subtracted, normalised, responses of MR units to cone-isolating stimuli (Top: non-opponent, *n* = 26; Middle: S-ON/L-OFF, *n* = 17; Bottom: L-ON/S-OFF, *n =* 7). **e, f** Mean ± SEM contrast response relationships (percentage variance in firing accounted for by the stimulus) of opponent (**e**; *n* = 24) or non-opponent (**f**, *n =* 26) MR cells for single opsin stimuli (left; classified according to response sign in **e** and strength in **f**) or for stimuli modulating both cone opsins in unison or antiphase (right). Data were analysed by two-way RM ANOVA with Sidak’s post-test; *, ** and *** represent *P <* 0.05, *P* < 0.01 and *P* < 0.001. **g** Proportions of MR and non-MR units exhibiting each response type and opsin preferences for all units of each class. Response type distributions compared by Fisher’s exact text. See also Additional file 3: Figure S3 for further data on non-MR cell responses

Having identified pretectal neurons that received input from ipRGCs, we next aimed to comprehensively define how their activity was modulated by cone input and determine whether any exhibited the colour-opponent behaviour observed previously in another major ipRGC target—the SCN [27]. To this end, starting with a polychromatic background stimulus that resembled a wildtype mouse’s experience of natural daylight (Fig. 2a), we adjusted the spectral composition to generate carefully calibrated pairs of stimuli designed to differ in apparent brightness for one or both cone opsins without significantly altering rod or melanopsin excitation (Fig. 2b; see also ‘Methods’ for further details). In particular, we generated four stimulus pairs where transitions between each element selectively modulated excitation of just L- or S-cone opsin in isolation, or both opsins in unison (‘L + S’) or in antiphase (‘L − S’). We then applied 0.25 Hz square-wave cycles of these cone-isolating stimulus pairs at a range of contrasts up to 75% Michelson (sevenfold change in apparent brightness).

The majority of MR units tested with these stimuli (*n* = 50/60) exhibited robust responses to at least a subset of the cone-isolating conditions tested (Fig. 2c–g). Moreover, a substantial proportion of these (*n* = 24/50) changed their firing rates in opposite directions when we selectively modulated L- or S-cone excitation in isolation; that is, almost half of these MR cells exhibited colour-opponency. Among this group, S-ON/L-OFF behaviour was substantially more common than the converse (*n* = 17 vs. *n* = 7 with L-ON/S-OFF responses); however, both classes of opponent MR cells usually exhibited a significant bias (on average ~ 3-fold larger responses) towards the excitatory (ON) component of the response (Fig. 2c, d, g).

We next inspected contrast response functions for ON and OFF responses among these colour opponent cells, by quantifying the percentage variance in firing rate accounted for by stimuli providing 15–75% contrast for the target cone population (see ‘Data analysis’ section in ‘Methods’ for further details). This analysis indicated, while OFF responses were reliably smaller at all tested contrasts (Fig. 2e; two-way RM ANOVA with Sidak’s post-tests), the sensitivity of ON and OFF responses was similar. Consistent with this behaviour, while these opponent MR cells were capable of responding to stimuli that modulated cone ‘illuminance’ without changing chromaticity (L + S stimulus), their responses were significantly larger when activation of the two cone opsins was modulated in antiphase to produce pronounced changes in colour (Fig. 2e; two-way RM ANOVA with Sidak’s post-tests). This chromatic response enhancement was evident even under the lowest contrast tested (15% Michelson).

Anatomically intermingled with the cell populations described above (Additional file 3: Figure S3), the remaining MR cells that exhibited robust responses to our cone-isolating stimuli lacked any clear evidence of opponent behaviour. In most cases, the responses of this group of cells were strongly biased towards one of the two opsins, with similar numbers of cells primarily driven by L-cone or S-cone opsin (Fig. 2g, *n* = 12 and *n* = 14 respectively). At the level of the population average then (Fig. 2d), achromatic (L + S) modulations in cone illuminance drove much more robust changes in firing than antiphasic (L − S) modulations (due to differential modulation of L- and S-biased subpopulations). By contrast, at the level of individual cells, responses to L + S stimuli tended to be only marginally elevated relative to those produced by L − S stimuli (Fig. 2e, two-way RM ANOVA with Sidak’s post-tests), consistent with the typically very weak responses driven by the ‘non-dominant’ opsin.

This diversity in the responses of pretectal MR units and, especially, the prevalence of cells exhibiting S-ON/L-OFF type colour opponent responses recapitulates our previous findings in the SCN [27], where retinal input comes almost exclusively via ipRGCs. By contrast, among the pretectal neurons identified here where we did not find clear evidence of melanopsin-dependent responses (Additional file 4: Figure S4), S-ON/L-OFF responses were significantly less common (Fig. 2g, *n* = 16/170, vs. 17/60 MR cells; Fisher’s exact test, *P* = 0.001) while the prevalence of L-ON/S-OFF and non-opponent cone-driven responses was similar (*P* = 0.136 and *P* = 0.446 respectively). Instead, across this group, there was a higher proportion of cells lacking detectable responses (*n* = 81/170 vs. 10/60 MR cells, *P* < 0.0001), indicating that either such cells primarily receive rod input or, perhaps more likely, that they are specialised to detect specific stimulus feature, e.g. motion or spatial contrast [46]. These differences in the relative proportions of response types held true also when we analysed data separately for non-MR cell populations (Additional file 4: Figure S4 g) designated ‘transient’ or ‘OFF’ but not the very rarely encountered group with sustained responses (where it is harder for us to definitively rule out a melanopsin contribution). Collectively then, alongside the recent identification of a subtype of mouse ipRGC (M5) with colour opponent input [28] and our earlier data [27], the present findings indicate a close association between the presence of melanopsin input and S-ON/L-OFF chromatic input.

To rule out the possibility that our assessment of cone preferences (and especially our inability to detect such responses in subsets of cells) simply reflected the relatively high background light intensity used, in a subset of experiments (*n* = 10 *Opn1mw*^*R*^ mice), prior to collecting the data reported in Fig. 2, we also applied identical cone-isolating stimuli but at a background 10× dimmer (ND1; intensity ~equivalent to sunrise/sunset). In fact, we found all cell groups tested displayed remarkably consistent responses at both background light intensities (Additional file 5: Figure S5a), barring a small number of cells that only responded under one of the two intensities (*n* = 12 and *n* = 6 responding only at ND1 and ND0 respectively from 133 cells tested). Response amplitudes were, however, modestly increased at lower irradiance (Additional file 5: Figure S5b, paired *t* test *P* < 0.0001). Since, for MR cells, peak firing rates were also at least nominally higher at the lower intensity (Additional file 5: Figure S5c), we suspect the slight reduction in response amplitude at higher irradiance reflects some degree of contrast adaption (since the higher irradiance also followed the lower in these experiments). Most importantly, however, the fundamental nature of the cone-dependent modulations in firing (opponent vs. non-opponent) was retained across all cell types.

#### Validation of cone-isolating stimuli

Having established the impact of cone-isolating stimuli on the activity of visually responsive neurons in the PON and surrounding pretectum, we next sought to determine the extent to which contributions from photoreceptors other than those specifically targeted by our stimuli could have influenced the results.

We first evaluated the possibility of a contribution from melanopsin. Since the cone-isolating stimuli we employed provided negligible melanopic contrast (< 6%, equivalent to a 0.05log unit change), well below the level required to evoke detectable responses [47], we considered it most unlikely that this inner retinal photoreceptor exerted any significant influence over the responses reported above. Consistent with that expectation, pretectal neurons recorded in *Opn1mw*^*R*^*;Opn4*^*−/−*^ mice under the same conditions behaved in an identical manner to those in mice with functional melanopsin: we readily identified both colour opponent and non-opponent neurons in the same proportions as in *Opn1mw*^*R*^ mice and those response preferences were stable when we changed irradiance (Additional file 6: Figure S6).

**Fig. 3:**
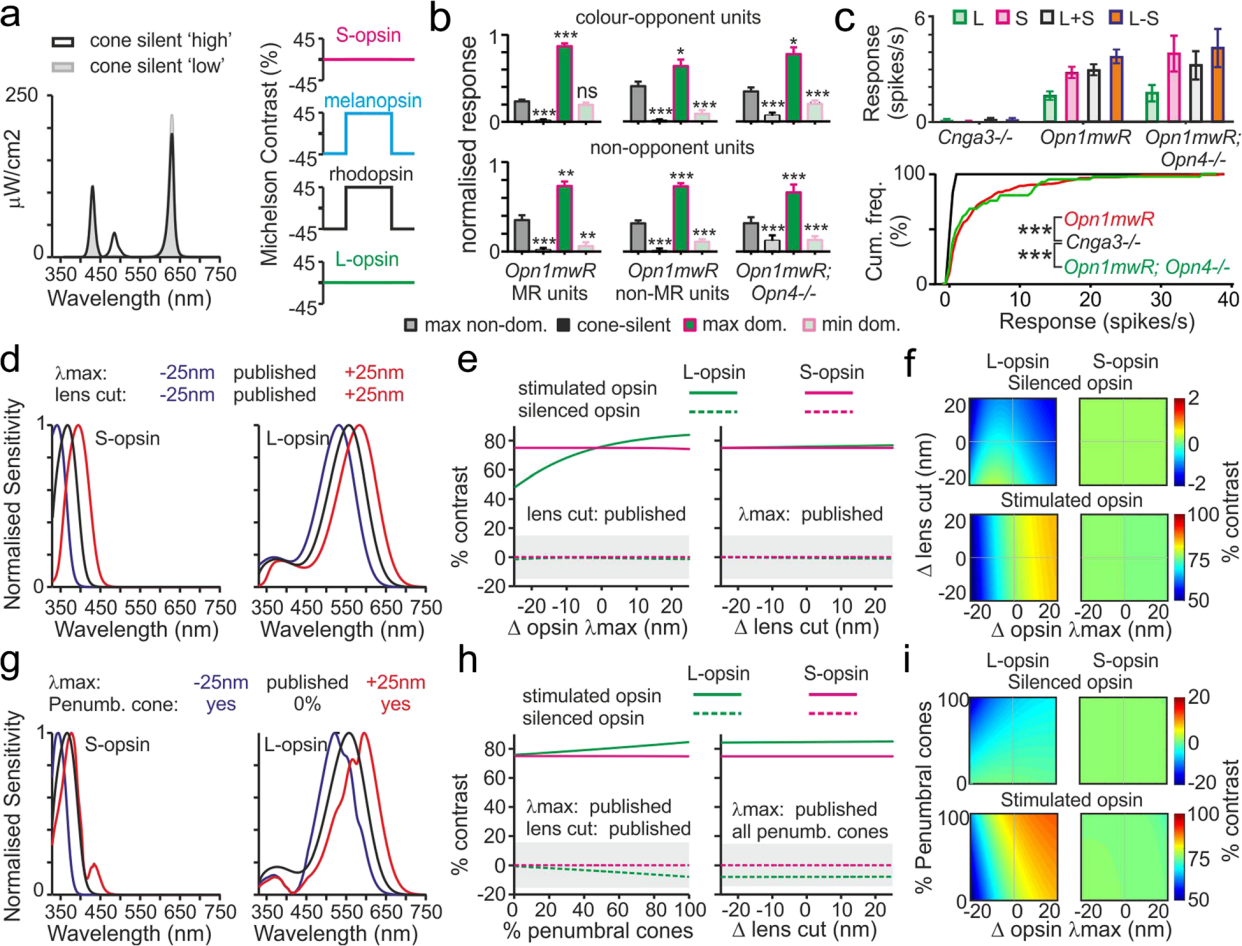
Validation of cone-isolating stimuli. **a** Left: Spectral power distribution of a ‘cone-silent’ stimulus pair designed to produce a large modulation in spectra but negligible cone contrast (< 0.05%). Right: calculated contrast produced by the cone-silent stimulus for each photoreceptor class. **b** Mean ± SEM responses of colour opponent and non-opponent MR and non-MR units in *Opn1mw*^*R*^ and *Opn1mw*^*R*^*;Opn4*^*−/−*^ mice (peak-trough modulation in firing normalised to largest cone-driven response), showing maximal responses to cone-isolating stimuli targeting the non-dominant or dominant opsin, 15% contrast stimuli targeting the dominant opsin and responses evoked by cone silent stimuli. Data were analysed by one-way RM ANOVA with Dunnett’s post-test (clockwise from top left *n* = 24, 25, 10, 13, 64 and 26). **c** Top: Mean ± SEM responses of *Cnga3*^*−/−*^, *Opn1mw*^*R*^ and *Opn1mw*^*R*^*;Opn4*^*−/−*^ neurons to 75% contrast cone-isolating stimuli; since (as expected) *Cnga3*^*−/−*^ lacked responses this analysis included all light-responsive cells identified in all genotypes (*n =* 24, *n* = 230 and *n* = 41 respectively). Bottom: cumulative frequency distribution for maximal response evoked by cone-isolating stimuli in the same populations of cells. Data were analysed by Kruskal-Wallis test with Dunn’s test for multiple comparisons. *, ** and *** indicate *P <* 0.05, *P <* 0.01 and *P <* 0.001 respectively; ns indicates *P* > 0.05. **d, g** Changes in cone opsin spectral sensitivity resulting from extreme deviations in peak sensitivity (λmax) and short-wavelength lens cut-off (**d**) or filtering via retinal vasculature (**g**; penumbral cones). **e**, **h** Changes in actual cone opsin contrast for L- and S-opsin-isolating stimuli (nominally providing 75% contrast) as a result of varying λmax, lens cut-off or contribution of penumbral cones. Shaded area represents minimal off-target contrast required to account for colour opponent responses identified. **f**, **i** Changes in cone-opsin contrast for L- and S-opsin-isolating stimuli as a result of varying both λmax and lens cut-off wavelength (**f**) or contribution of penumbral cones (**i**). Colour axes are symmetrical about the target contrast (note difference in scaling between panels)

Since our cone-isolating stimuli also provided negligible contrast for rods (1.7 and 3.3% for L- and S-opsin-isolating stimuli respectively), we also considered a significant contribution from rods most unlikely. Indeed, the rod contrast associated with our cone-isolating stimuli was lower than previously reported thresholds for rod-based responses under light adaptation [41, 47–49]. Nonetheless, to more directly rule out the possibility that our cone-isolating stimuli drove responses via photoreceptors other than those targeted, we next evaluated the impact of a stimulus pair designed to produce no detectable contrast for either cone opsin (< 0.05% Michelson contrast), while presenting a significant change in rod and melanopsin activation (Fig. 3a; ‘cone-silent’; 45 and 43% contrast for rod and melanopsin respectively).

Importantly, we found that across all classes of visually responsive pretectal neurons (colour-opponent and non-opponent, MR and non-MR) in both *Opn1mw*^*R*^ and *Opn1mw*^*R*^*;Opn4*^*−/−*^ mice, this cone-silent stimulus produced consistently negligible modulations in the firing rate. Indeed, changes in firing associated with presentation of this stimulus were in all cases significantly lower than those evoked even by the weaker of the two (non-dominant) single-cone stimuli for that cell (Fig. 3b; one-way RM ANOVAs with Dunnett’s post-tests, all *P* < 0.001). These data thus rule out the possibility that the responses we ascribe to cones in fact originate with rods and/or melanopsin.

To further confirm our interpretation above, we also evaluated the impact of cone-isolating stimuli on pretectal neurons in *Cnga3*^*−/−*^ animals. As expected, given the absence of a critical component of the cone-phototransduction cascade, presentation of these cone-isolating stimuli did not produce detectable responses in *Cnga3*^*−/−*^ cells (Fig. 3c, *n* = 24 visually responsive neurons reported in Fig. 1 and Additional files 1 and 2). As such, statistical comparison of the maximal response of each cell to any of the cone-isolating stimuli we applied revealed a pronounced difference relative to those of cells from both *Opn1mw*^*R*^ and *Opn1mw*^*R*^*;Opn4*^*−/−*^ mice (Fig. 3c, Kruskal-Wallis test with Dunn’s multiple comparisons, both *P* < 0.0001; analysis based on all visually responsive neurons, regardless of response to cone-isolating stimuli). In addition, equivalent analyses to those reported above (Fig. 3b, c) for subsets of cells tested at 10-fold lower irradiance produced identical results (Additional file 7: Figure S7); in no case did we observe any evidence consistent with any significant rod or melanopsin intrusion.

To complement the important control experiments described above, we also considered whether variations in cone opsin sensitivity or prereceptoral filtering, relative to those assumed when generating our stimuli, might result in off-target cone-driven responses under conditions designed to silence one or the other class. In particular we were keen to rule out the possibility that the colour-opponent responses we detected might instead have reflected some ‘overshoot’, such that stimuli expected to be silent in fact induced detectable negative contrast responses in that cone population.

To this end, we first modelled the impact of very substantial variations in the cone opsin peak sensitivity (λmax) and/or in the short-wavelength cut-off imposed by the mouse lens (Fig. 3d–f; in each case ± 25 nm—far beyond any reasonable estimate of errors associated with the published values). In fact, we found that even unfeasibly large deviations from the assumptions inherent in our stimulus design had negligible effects, particularly in the case of predicted S-opsin responses which were effectively insensitive to these manipulations. Moreover, while the potential impact on L-opsin-driven responses was slightly larger, stimuli expected to be L-cone silent continued to present negligible contrast even following the most extreme deviations in both lens transmission and opsin sensitivity (Fig. 3c; <±2% contrast).

We also considered other sorts of prereceptoral filtering. In humans, under certain circumstances, stimuli expected to be cone silent generate a percept of the retinal vasculature [50], due to differential stimulation of cones that lie in the shadow of blood vessels (penumbral cones). Given the very large size of mouse RGCs (especially ipRGCs; [43]), it is most unlikely that any of the pretectal units we sampled from could receive input exclusively from penumbral cones. Nonetheless, we assessed the possible impact of such a phenomenon, by modelling the effect of varying contributions of penumbral cones alongside deviations in L- and S-opsin λmax (Fig. 3g–i). Again, we found that neither mechanism (singly, in combination or even with the addition of variations in lens cut-off) significantly altered predicted S-opsin-driven responses. Moreover, while including penumbral cones could nominally increase off-target contrast for stimuli expected to be L-cone silent (Fig. 3h), even in the implausible case that a cell received input exclusively from penumbral cones, the expected magnitude of this effect (< 8% negative L-opsin contrast) was far too small to account for the colour opponent responses we observed (where responses driven by the weaker of the two cone opsins were at least as large as those driven by 15% contrast stimuli applied to the stronger; see Fig. 3b). Indeed, our further analysis revealed that for any noticeable interference due to off-target effects to occur, not only would input have to come from an unfeasibly high proportion of penumbral cones but also that L-opsin would have to have a far more short-wavelength λmax than any reasonable expectation (Fig. 3i). Our experimental data rule out this most unlikely possibility. Hence, additional modelling indicated that any deviation in cone-opsin λmax sufficient to result in detectable off-target effects would result in an even larger unintended contrast for our cone-silent stimulus (Additional file 7: Figure S7c). As reported above (Fig. 3b), this stimulus did not produce detectable responses.

**Fig. 4:**
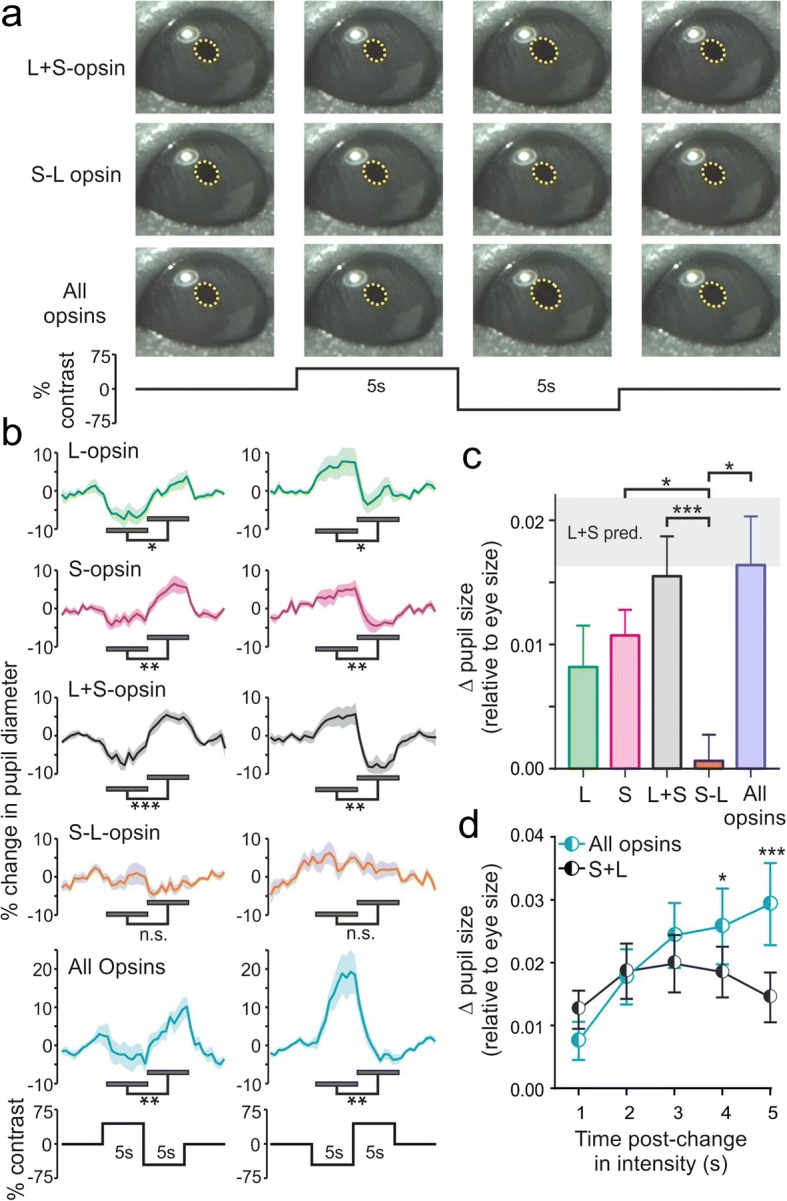
Signals from both cone types and melanopsin combine in an additive manner to drive pupil constriction. **a** Representative images of mouse consensual pupil responses evoked by stimuli modulating just L- and S-opsin (in unison or antiphase) or all photoreceptors at 75% contrast. **b** Mean ± SEM percent change in pupil diameter across 10 mice for cone-isolating and all photoreceptor stimuli applied as square-wave modulations at two different polarities (two to four trials/mouse). Mean pupil size between ‘bright’ and ‘dim’ stimulus phases were compared by paired *t* tests. **c** Mean ± SEM change in pupil diameter between ‘bright’ and ‘dim’ stimulus phases (averaged across both stimulus polarities and normalised according to eye size) from animals contributing to panel **b**. Data were analysed by one-way RM ANOVA with Holm-Sidak’s multiple comparisons test. Shaded area represents mean ± SEM change in pupil size predicted from a linear sum of responses to L and S-opsin-isolating stimuli. **d** Mean ± SEM difference in pupil diameter between ‘bright’ and ‘dim’ stimulus phases (processed as in **c**) at various timepoints following the stimulus transition. Data were analysed by two-way RM ANOVA with Sidak’s post-test. For **b**–**d**, ***, **, * and n.s. indicate *P <* 0.001, *P* < 0.01, *P* < 0.05 and *P >* 0.05 respectively

**Fig. 5:**
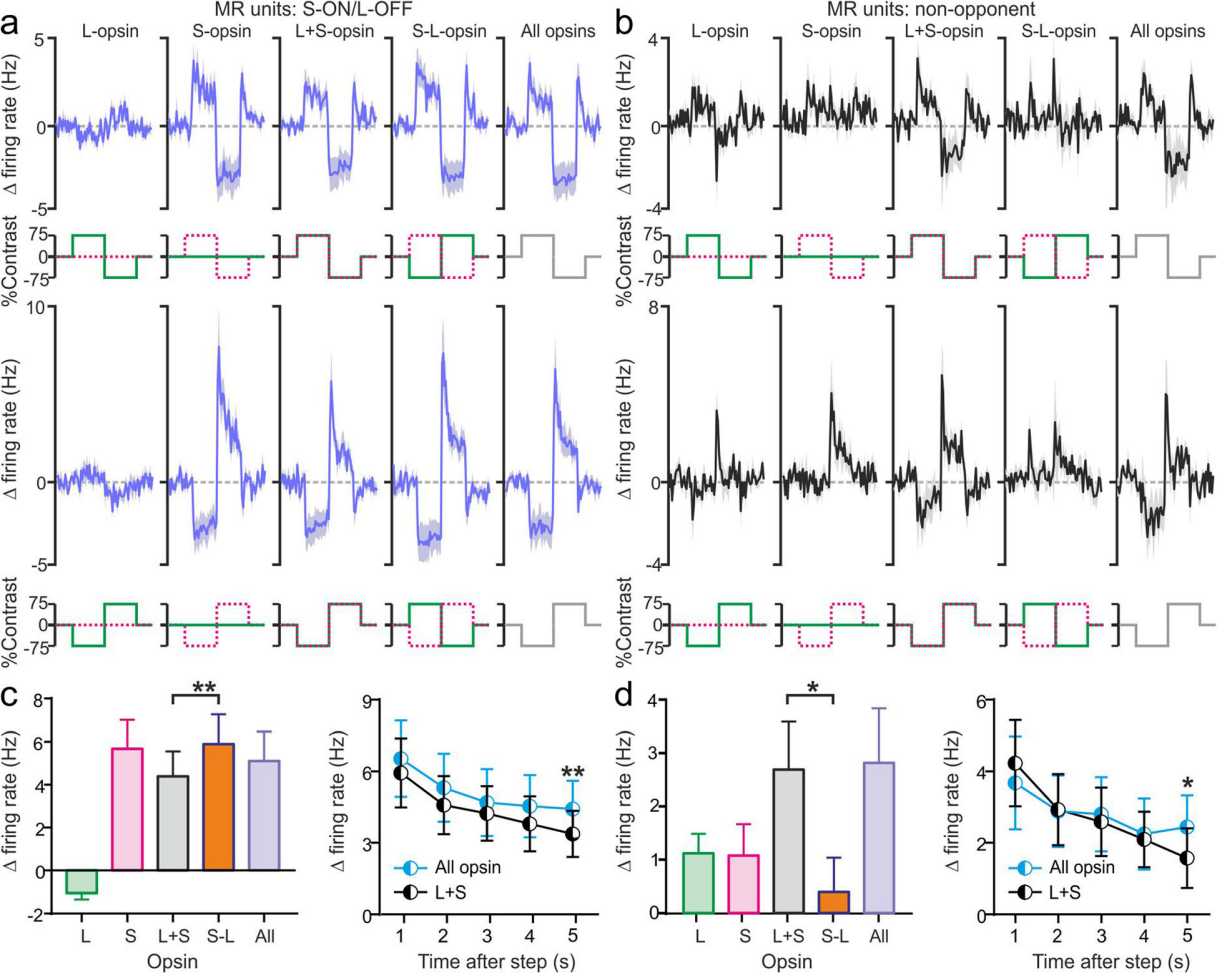
Opponent and non-opponent melanopsin-responsive units are readily identifiable under the stimulus paradigm used for pupillography. **a**, **b** Mean ± SEM baseline subtracted responses of S-ON/L-OFF (**a**; *n =* 7) and non-opponent (**b**; *n =* 9) MR units to 75% contrast cone isolating and all opsin stimuli delivered as for pupillography (Fig. 4). The sample tested here (*n =* 5 *Opn1mw*^*R*^ mice) did not include any L-ON/S-OFF MR units. **c**, **d** Left: Mean ± SEM change in firing between ‘dim’ and ‘bright’ stimulus phases for all stimuli (averaged across full 5 s phase and both stimulus polarities) for S-ON/L-OFF (**c**) and non-opponent units (**d**) as above. Data analysed by Freidman’s ANOVA with Dunn’s multiple comparison test. Right: Mean ± SEM change in firing between ‘dim’ and ‘bright’ stimulus phases for 75% contrast stimuli targeting L + S cone opsins or all photoreceptors as a function of time since contrast step (averaged across both stimulus polarities as above). Data analysed by two-way RM ANOVA with Sidak’s post-tests. * and ** = *P* < 0.05 and 0.01 respectively

In sum then, we can exclude the possibility that responses originating with rods, melanopsin or unintended stimulation of cones interfere with our conclusions regarding cone-specific influences on pretectal neurons. Additional analysis reported below (Figs. 4, 5 and 7) provides further confirmation that our stimuli work as expected under a variety of different conditions, such that stimuli with very different spectral compositions but similar predicted photoreceptor contributions consistently produce equivalent responses.

#### Influence of cones on mouse pupil responses

Insofar as our data above reveal that cone inputs provide high-amplitude chromatic or illuminance signals to subsets of pretectal MR units, we next investigated the extent to which these distinct sources of visual information were important for regulation of the primary physiological output known to be under PON control—the pupil. For these experiments, we used the same 75% contrast cone-isolating stimuli described above and, based on the previously reported dynamic range for mouse pupil responses [24, 25, 51] and our data showing moderately enhanced cone-based responses (Additional file 5: Figures S5 and Additional file 6: Figure S6), presented these at the lower of the two backgrounds tested (10-fold dimmer than that in Fig. 2b, corresponding to typical irradiance at sunrise/sunset). Since previous data reveal important contributions of melanopsin and rods to mouse pupil responses [25, 51, 52], we also aimed to understand how any influence of cones compared with that originating with those other photoreceptor classes under our conditions. As such we included an additional stimulus (‘all opsin’) that provided a spectrally neutral modulation in light intensity providing 75% contrast for every class of mouse photoreceptor. Finally, to account for the more sluggish nature of pupil responses relative to PON neuronal activity, we extended our stimulus presentation here to 5 s at each phase and presented these as single cycle ‘bright-dim’ and ‘dim-bright’ modulations (stimuli interleaved and order randomised between animals; 2–4 trials/stimulus/animal). This allowed us to test all stimuli multiple times in a single animal while avoiding any confound due to contrast adaptation. We then went on to record consensual pupil responses to these stimuli from awake gently restrained mice (Fig. 4a; *n* = 10 *Opn1mw*^*R*^ mice).

Across both stimulus polarities tested, we reliably observed significantly larger pupil diameters during ‘dim’ vs. ‘bright’ phases when the stimuli selectively modulated either L- or S-cone opsin in isolation (Fig. 4b; paired *t* tests all *P* < 0.05). The same was also true when we modulated just L + S opsin or all opsins in unison to produce achromatic changes in illuminance. By contrast, stimuli that modulated L and S-opsin in antiphase (and thereby produced large changes in colour without changing illuminance) failed to evoked significant changes in pupil diameter (Fig. 4b; paired *t* tests; *P* = 0.23 and 0.32 for positive-negative and negative-positive modulations respectively).

As expected, baseline pupil diameter did not vary significantly between any of the stimulus conditions (one-way RM ANOVA, *P* = 0.11) nor did the difference in pupil diameter between dim and bright phases vary as a function of stimulus polarity (two-way RM ANOVA, *P* > 0.05 for polarity and interaction). As such, we averaged ‘bright’-‘dim’ change in pupil diameter across both stimulus polarities for comparisons between stimuli (Fig. 4c). This analysis confirmed that modulations in pupil size evoked by achromatic L + S opsin modulations were significantly larger than those produced by chromatic, antiphasic modulations in the two cone opsin classes (Fig. 4c; one-way RM ANOVA with Sidak’s post-test, *P* < 0.001). Moreover, the change in pupil size evoked by L + S cone opsin modulations was not significantly different from that predicted by a simple linear sum of the responses to stimuli modulating just L- or S-opsin in isolation (Fig. 4c, paired *t* test, *P* = 0.49). Collectively then, these data indicate that, despite the presence of large numbers of chromatic MR units in the mouse PON, cone-derived chromatic signals do not noticeably contribute to regulation of the mouse pupil. Instead signals from L- and S-opsins combine in an additive manner to drive pupil constriction.

To rule out the possibility that this lack of chromatic modulation of the mouse pupil simply reflected differences in the temporal waveform of the stimuli between those used for electrophysiology and pupillography, we also evaluated neurophysiological responses of a subset of pretectal MR units under the latter stimulus paradigm. As expected, these recordings confirmed that both colour opponent and non-opponent MR neuronal responses remained readily identifiable under the conditions used for pupillography (Fig. 5a, b; *n* = 7 S-ON/L-OFF and 9 non-opponent neurons from 5 *Opn1mw*^*R*^ mice). A similar finding was true also for pretectal neurons in *Opn1mw*^*R*^*;Opn4*^*−/−*^ mice tested under these conditions (Additional file 8: Figure S8a,b).

In addition to demonstrating that cones exert an additive rather than chromatic influence on mouse pupil regulation, the analysis above also highlighted another important feature of pupil control under our experimental conditions; when comparing the average change in pupil size between ‘bright’ and ‘dim’ across the full 5-s stimulus duration, there was no detectable difference between stimuli that modulated just L- and S-opsin vs. those that which modulated all mouse opsins equally (Fig. 4c, one-way RM ANOVA with Sidak’s post-test, *P* = 0.85). This indicates that, under the photopic conditions studied here, pupil responses to relatively small/rapid changes in illumination are dominated by cone rather than rod and/or melanopsin-derived signals.

To explore this conclusion in more detail, we next analysed the difference in pupil size between dim and bright stimulus phases of L + S vs. all-opsin stimuli as a function of time-post stimulus transition (Fig. 4d). This analysis in fact revealed a progressive divergence between pupil responses to the two stimuli, with both types of stimuli producing equivalent responses for the first 3 s and all-opsin modulations producing larger changes in pupil size across the later components of the stimuli (Fig. 4d; two-way RM ANOVA with Sidak’s post-tests). Equivalent effects were also observed in the electrophysiological responses of MR neurons (Fig. 5c, d). This gradual appearance of a cone-independent influence on pupil responses and PON neurophysiological activity closely matches that expected from melanopsin, based on pupil data reported for melanopsin only and melanopsin knockout animals [25, 51, 52] and electrophysiological recordings from the PON and other ipRGC target regions [11, 16, 27, 40, 44, 47, 53, 54]. By contrast, rod-driven pupil and electrophysiological responses are much faster [25, 41, 47] (see also data from *Cnga3*^*−/−*^ mice presented in Fig. 1 and Additional file 1: Figure S1 and Additional file 2: Figure S2). Importantly then, the close similarity between initial components of L + S and all-opsin responses observed here (Fig. 4d and Fig. 5c, d) rules out any overt contribution from rods under the conditions we employed. Also consistent with our interpretation that the divergence in response to these two stimuli at later timepoints originates with melanopsin, this feature was lacking from the neurophysiological responses of *Opn1mw*^*R*^*;Opn4*^*−/−*^ cells tested under these conditions (Additional file 8: Figure S8c,d). Further data reported below confirm and extend this conclusion under a variety of different conditions (Fig. 7 and associated additional files).

These data therefore confirm additive contributions from both cone types and melanopsin to pupillary control, with cones dominating early components of the responses and melanopsin becoming a progressively more important contributor under extended exposure.

### Temporal properties of cone influences on melanopsin-responsive neurons

Given our data above, we next sought to better understand the temporal properties of cone inputs to MR units in the PON. In particular, a number of previous studies have provided evidence that S-cone contributions to ipRGC-dependent physiological responses (including PON neuronal activity) may exhibit temporal properties that are distinct from those originating with longer-wavelength-sensitive cones [11, 30, 55]. Here then, we examined in more detail the temporal tuning properties of cone inputs to MR cells by applying cone-isolating stimuli as sinusoidal oscillations across a range of temporal frequencies (0.025 to 5 Hz, 75% contrast, ND0).

**Fig. 6:**
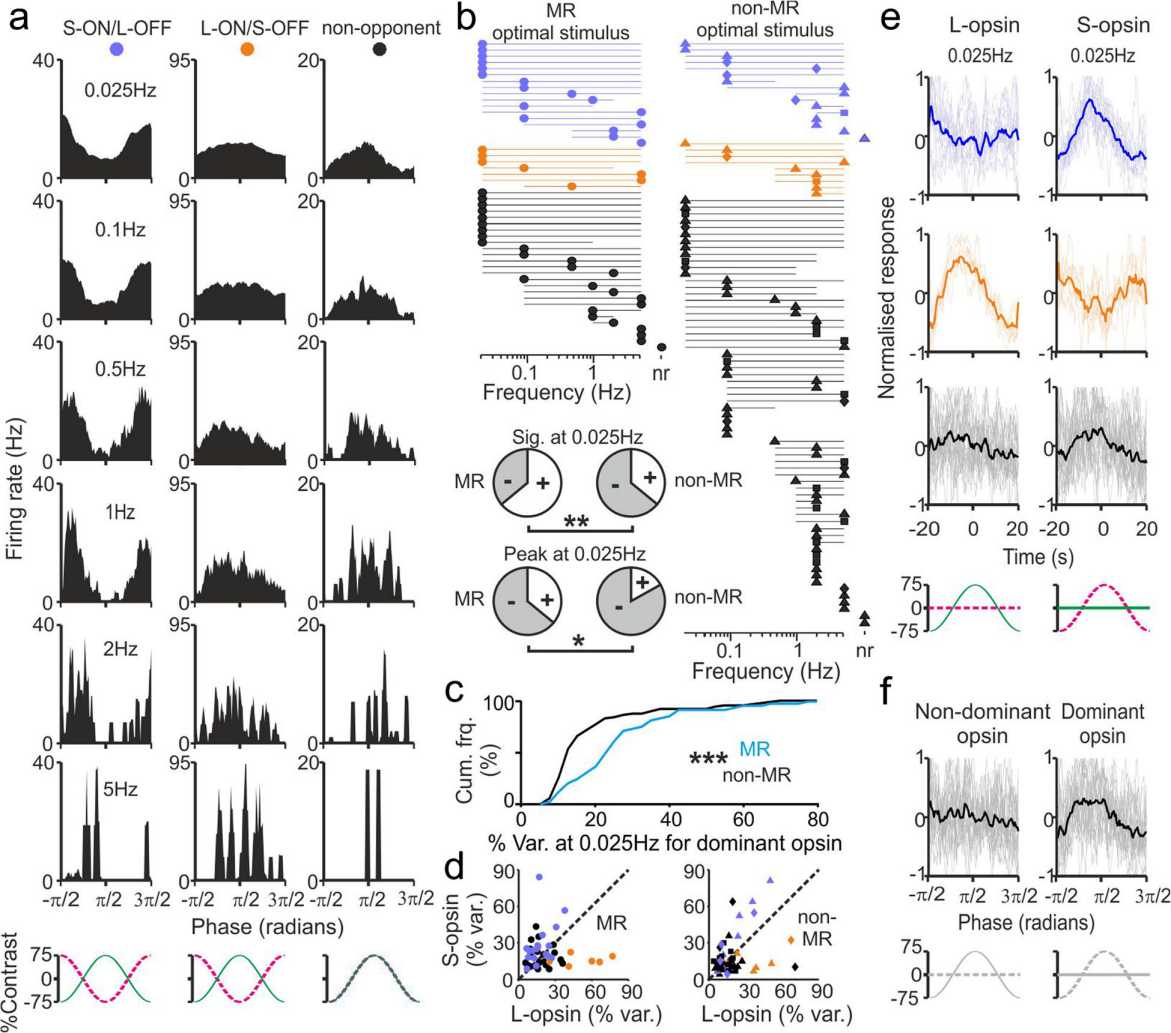
Cone inputs to melanopsin-responsive cells provide information about gradual changes in illuminance or colour. **a** Example peristimulus firing rate histograms for three MR neurons tested with sinusoidal oscillations of their optimal cone stimulus type (L − S modulation for chromatic units and L + S stimulus for the non-opponent unit—right) at 75% contrast and varying temporal frequency. **b** Population data showing, for each responding MR cell and non-MR cell (as defined in Fig. 2), the range of frequencies that drove significant modulations in the firing rate and preferred temporal frequency for optimal cone stimuli (indicated by start and endpoints of each line and symbols respectively; assessed by *χ*^2^-periodogram, see ‘Methods’ for further details). n.r. indicates no significant response at any frequency. Inset pie charts show proportions with significant response and strongest responses at 0.025 Hz. Data analysed by Fisher’s exact test, * and ** = < 0.05 and 0.01 respectively. **c** Cumulative distribution for MR and non-MR (same populations as in **b**) responses to 75% contrast stimuli applied to dominant cone opsin (quantified as percentage variance in firing rate accounted for by the 0.025-Hz stimulus). Data analysed by Kolmogorov-Smirnov test, *** = *P* < 0.0001. **d** Quantification of responses for low-frequency L- and S-opsin-driven responses across each responding MR cell (quantified as in **c**). **e** Normalised responses to low-frequency L- and S-opsin-isolating stimuli for each class MR neuron; thin traces represent individual cells, thicker lines represent population average. **f** Data for achromatic MR units (bottom panels of **d**) sorted according to preferred and non-preferred opsin based on analysis in Fig. 2

We first examined the responses of MR cells to their ‘optimal’ stimulus type (L − S and L + S stimuli for colour-opponent and non-opponent cells respectively) and found that the majority of MR neurons that responded to square-wave modulations also exhibited significant responses across a wide range of sinusoidal stimulus frequencies (Fig. 6a, b). Indeed, substantial numbers of MR cells continued to track modulations in cone-derived illuminance or chromatic signals even at the lowest temporal frequency tested (Fig. 6b, *n* = 32/50). This observation applied similarly to both colour-opponent and non-opponent MR neurons (*n* = 18/24 and 14/26 respectively; Fisher’s exact test, *P* = 0.15). By contrast, non-MR cells (Additional file 9: Figure S9) generally showed significant modulations in the firing rate over a narrower range of temporal frequencies, with a much lower proportion of cells capable of tracking the lowest temporal frequency we tested (Fig. 6b; *n* = 32/89 cells that responded to square-wave stimuli, Fisher’s exact test, *P* = 0.002 vs. MR units). Similarly, while a substantial proportion of MR cells in fact exhibited the most robust modulations in firing rate at the lowest frequency tested (*n* = 18/50; quantified as percent variance in firing rate explained by a 0.025-Hz stimulus), this was less commonly observed across non-MR cells (*n* = 15/89; Fisher’s exact test *P* = 0.013). Collectively then, these data support previous suggestions that cone inputs to ipRGCs are unusually sustained [14, 15, 53] and indicate that MR neurons in the pretectum can use cone inputs to track even quite gradual changes in illuminance or colour.

We next examined whether the L- and S-opsin-driven responses of MR units differed in their ability to track low-frequency modulations. In line with the data reported above, we found that low-frequency sinusoidal modulations of the cone opsin class to which that cell was biased (as determined from response to square-wave stimuli, Fig. 2) reliably evoked robust responses in a significantly larger population of MR vs. non-MR neurons (Fig. 6c; Kolmogorov-Smirnov test, *P* < 0.0001). Further analysis classes confirmed that, as expected based on data presented above (Fig. 2), there were near equivalent proportions of both MR and non-MR neurons that preferentially responded to low-frequency stimuli targeting L- or S-opsin (Fig. 6d; MR: *n* = 22 L-biased vs. 28 S-biased, non-MR: 38 vs. 51; Fisher’s exact test vs. equal proportions—both *P* > 0.05). Hence, for low temporal frequency stimuli, colour opponent cells almost always exhibited more robust variations in the firing rate when we modulated the opsin providing the excitatory/ON component of their response (Fig. 6e), whereas subsets of achromatic cells preferentially responded to low-frequency L- or S-opsin-isolating stimuli (Fig. 6d, e).

#### Relationship between cone and melanopsin inputs

The data described above indicate that cone inputs exert a powerful influence over MR-cell firing activity, even for relatively modest and gradual changes in illumination (sevenfold variation in intensity over 40 s). Nonetheless, data presented earlier in the manuscript (Figs. 1, 4 and 5) provide clear evidence for a progressive transition between cone- and melanopsin-derived influences on pretectal MR cell activity and pupil responses following extended exposure. We next then sought to more comprehensively define the relative influence of cones and melanopsin on MR-cell firing in response to the smaller, dynamic changes in light intensity a mouse might encounter during exploratory activity close to dawn and dusk.

For this analysis, we compared responses to square-wave or sinusoidal stimuli that selectively modulated L- and S- opsin in unison (data in Figs. 2 and 6), with equivalent stimuli that modulated all photoreceptor classes equally (all-opsin). As discussed above, in principle, any difference in the response to these stimuli could reflect a contribution of either rods or melanopsin. However, the relatively high background light intensity employed for the experiments should substantially impair any rod-mediated responses (Fig. 2b; ND0; 15.2 log rod effective photons). Indeed, the related analyses reported earlier in the manuscript at 10-fold lower irradiance (Figs. 4 and 5) certainly did not reveal any evidence of significant rod contributions.

**Fig. 7:**
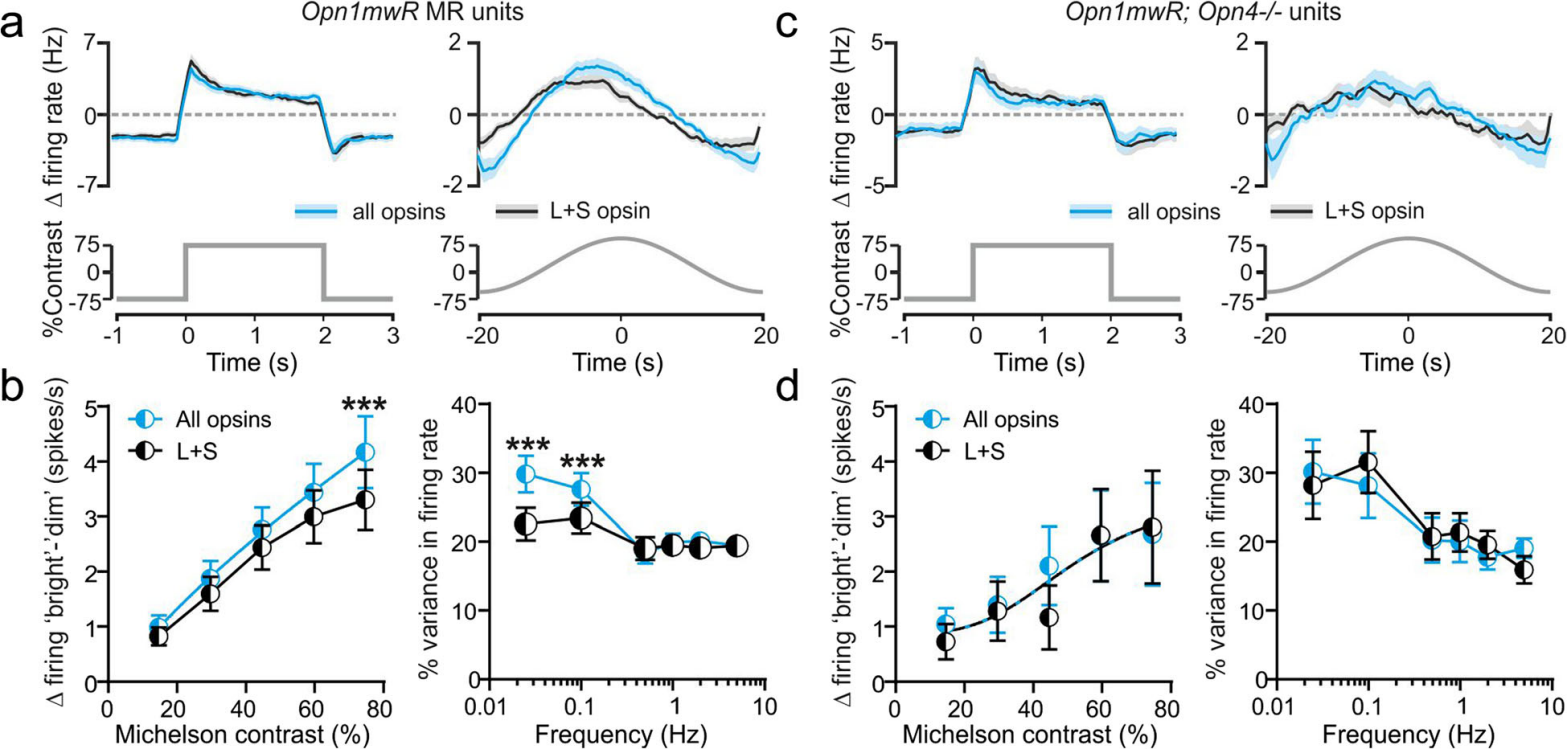
Contributions of melanopsin to pretectal neuronal responses. **a**, **c** Mean ± SEM responses of *Opn1mw*^*R*^ MR units (**a**; *n* = 50) and *Opn1mw*^*R*^*; Opn4*^*−/−*^ cells (**c**; *n =* 23) to rapid (0.25 Hz square wave; left) or gradual (0.025 Hz sinusoid; right) spectrally neutral stimulus modulations (all opsins) and stimuli targeting just L- and S-cone opsin (75% contrast). **b**, **d** Contrast (left) and temporal frequency (right) tuning curves for *Opn1mw*^*R*^ MR (**b**) and *Opn1mw*^*R*^*; Opn4*^*−/−*^ (**d**) responses to all opsin and L + S-opsin-isolating stimuli (as above). For contrast response analysis, data points represent difference in mean firing rate during the last 400 ms at ‘bright’ vs. ‘dim’ stimulus phases. For temporal frequency analysis, data points represent the % variance in firing rate accounted for the stimulus. In both cases, data analysed by two-way RM ANOVA with Sidak’s post-tests. *** = *P <* 0.001

As expected then, given the relatively sluggish nature of melanopsin responses, across all MR units that reliably responded to cone-isolating stimuli (*n* = 50), changes in the firing rate evoked by rapid L + S opsin-targeted stimuli (0.25 Hz square wave) were in fact remarkably similar to those produced by equivalent stimuli targeting all opsin classes (Fig. 7a). Nonetheless, when comparing the difference in firing rates between bright and dim stimulus phases, we found that by the last 400 ms at each phase there was a progressive divergence in the contrast response functions for the two stimuli such that, at the highest contrast, responses were greater for the all opsin stimuli (Fig. 7b; two-way RM ANOVA with Sidak’s post-test, *P* < 0.05). This divergence in responses between stimuli that modulate both cone opsin types with or without also engaging melanopsin became even more pronounced when we examined responses to low temporal frequency modulations (Fig. 7a). Indeed, comparison of the temporal frequency tuning relationship for L + S and all opsin stimuli across MR units revealed that, whereas responses were identical for frequencies of 0.5 Hz and greater, there was a substantial enhancement in responses at the two lowest frequencies tested (Fig. 7b, two-way RM ANOVA with Sidak’s post-tests, both *P* > 0.001).

Although the selective response enhancement for all opsin stimuli within a specific temporal domain matched well what one would predict for melanopsin, we next sought to confirm this was indeed the case. As such, we first performed the same analysis described above for the other classes of pretectal neurons encountered in *Opn1mw*^*R*^ mice that lacked conclusive evidence of melanopsin input (non-MR). As expected, we did not observe any significant response enhancement for all opsin stimuli across any tested contrast, temporal frequency or cell class (Additional file 10: Figure S10; two-way RM ANOVA with Sidak’s post-tests, all *P* > 0.05). We also compared the responses of pretectal neurons in melanopsin knockout animals (*n* = 23 *Opn1mw*^*R*^*; Opn4*^*−/−*^ cells responding at ND0) under the same conditions. Again, we observed no significant differences between L + S and all opsin stimuli at any contrast or temporal frequency (Fig. 7c, d; two-way RM ANOVA with Sidak’s post-tests, all *P* > 0.05). Finally, we performed a similar analysis of contrast response functions for units tested with square-wave stimuli at ND1 (Additional file 11: Figure S11) which again confirmed that *Opn1mw*^*R*^*; Opn4*^*−/−*^ cells exhibited identical responses to L + S and all-opsin stimuli while *Opn1mw*^*R*^ MR unit responses for these stimuli diverged in a manner equivalent to that shown at higher irradiance.

In sum, the analyses described above support our view that the appearance of a cone-independent component of the MR cell responses at high-contrast/low temporal frequency conditions reflects the contribution of melanopsin photoreception. Collectively then, these data indicate that, in line with previous suggestions [26], for the relatively small and rapid variations in light intensity/spectral composition most commonly encountered during exploratory activity during the day [53], MR neuronal responses are primarily driven by cones. By contrast, melanopsin signals become increasingly important for tracking slower/larger changes in irradiance, such as those encountered across twilight or when moving between areas with substantial differences in access to natural light.

## Discussion

Consistent with previous suggestions that chromatic signals may be of particular relevance for non-image-forming visual responses [33], here we show a substantial convergence of melanopsin and cone-dependent colour-opponent input across PON/pretectal neurons. Surprisingly, however, chromatic signals do not detectably impact on the best known physiological output under PON control—the pupil light reflex. Below we discuss the significance of these findings for understanding the roles and properties of ipRGCs and for sensory control of the pupil.

### Origins of cone-based responses in melanopsin-responsive neurons

The ipRGCs provide a major but not exclusive source of input to the PON [35] and the wider pretectum receives substantial input from non-melanopsin-expressing RGCs (c.f. [36, 43]). As such, the influence of cones on MR neurons described here need not directly recapitulate those of ipRGCs. Nonetheless, we here find that cone inputs to MR units (both colour-opponent and non-opponent) are characteristically more sustained than among non-MR cells, consistent with previous comparisons of cone influences on ipRGCs vs. other RGC types [15]. We also find that colour opponent responses are particularly common among MR cells in the PON, whereas such properties are significantly less so across PON cells that lack evidence of melanopsin input (although not altogether absent) and are very rare at the level of RGCs in general [56].

Of particular note here then, while an earlier investigation [15], failed to find evidence of colour opponency at the level of mouse ipRGCs, recent data indicate that a particular subtype of ipRGC (M5) displays the S-ON type colour opponency [28] which we commonly find in PON MR neurons (and previously found also in the SCN; [27]). One plausible origin for the colour opponency we observe in PON MR units then is that it is directly inherited from M5 cells. Since the responses of M5 cells are believed to rely on a specific S-ON bipolar cell, this mechanism cannot directly account for the smaller subset of MR units in the pretectum that exhibit the opposite (L-ON/S-OFF) colour preference, however, suggesting an alternative or additional origin.

Although ipRGCs with S-OFF properties have been identified in primates [29], they have yet to be observed in mouse retina. Nonetheless, one previous study has provided evidence that a subset of alpha-ganglion cells (and therefore potentially M4 ipRGCs; [57]) exploit the gradient in mouse cone opsin expression [58] to generate chromatic responses [59]. Such a mechanism could generate either S-ON or S-OFF type chromatic responses. Accordingly, the properties of PON MR units observed here could arise either via input from M4 cells or a combination of M4 and M5 ipRGCs. Either way, the proposed source of colour-opponency involves a centre-surround mechanism such that the chromatic influence is most apparent for stimuli larger than the cells receptive field centre. Centre-surround opponency of this type generally seems to result in stronger centre-driven ON vs. surround-driven OFF responses, which matches the bias we observe also in PON MR units with colour opponent responses. It should be noted, however, that while both M4 and M5 cells are known to project to the visual thalamus [28, 60] it is so far unclear whether any provide input to the PON. As such, it remains possible that the colour opponent responses we observe centrally arise via some alternative mechanism (e.g. involving central processing or convergent input from multiple RGC types). A similar situation is true also for the non-MR colour opponent neurons we observed. Such responses could in principle be directly inherited from one or more RGC types that do not express melanopsin but we cannot exclude the possibility that they instead arise via central processing. We do however note that the temporal tuning of cone-opponent input to MR and non-MR units differs (Fig. 6b), suggesting that the retinal/central mechanisms that generate their opponency also differ.

Although, to our knowledge, there have been no other investigations of chromatic processing in mouse brain regions involved in NIF visual responses, a final point to note here is that previous studies have suggested a qualitative difference in types of visual information provided by S- vs. M/L-cone opsin [11, 55]. In particular, it has appeared that S-opsin-driven responses are more sustained/better able to track lower temporal frequency events. Our data reveal that, in fact, both cone opsin types can readily track low-frequency stimuli, but that individual cells generally display strong preferences towards just one class. In aggregate then, our present data support the previous suggestion that mouse PON neurons are better able to track gradual changes in light at shorter wavelengths [11] but suggest this is not because of any fundamental difference in the temporal properties of S- vs. L-cone pathways that impinge on ipRGCs. Instead, it seems simply that, because both cone types show significant sensitivity in the UV-blue region of the spectrum (e.g. see Fig. 1a), shorter-wavelength stimuli recruit more neurons (i.e. both S- and L-cone biased non-opponent cells and S-ON/L-OFF colour opponent cells).

#### Physiological roles of melanopsin-responsive neurons

The best established function of the PON is pupil regulation [46]. Previous investigations of pupil control in mice have revealed a strong reliance on ipRGC input [35, 61, 62], with melanopsin required for maximal consensual pupil constriction under high light levels [51]. By contrast, the role of outer retinal signals in mouse pupil regulation has remained more controversial, with two previous assessments concluding that the sensitivity of pupil responses were defined by cones [24] or rods [25]. In fact, the different conclusions of these studies appear primarily methodological in origin, with a parsimonious interpretation being that both classes of photoreceptor can contribute to pupil regulation. Certainly, the more recent of these two studies shows clearly both that rods can drive very pronounced and sustained pupil constriction and also that ‘cone-only’ animals retain some (more transient) pupil responses [25].

Although we did not specifically set out to address the role of rods here, collectively our data clearly support the view that all known classes of photoreceptor provide input to PON neurons, including recordings from mice lacking functional cones (which provide clear evidence of rod responses; Fig. 1 and Additional file 1: Figure S1 and Additional file 2: Figure S2). As such, it seems relatively unsurprising that rods should define the sensitivity of pupil constriction under appropriate conditions [25]. Nonetheless, pupil constriction in rod-only animals reaches its maximal level at quite low light intensities (equivalent to those encountered around civil twilight) whereas pupil responses in wildtype mice require 100–1000 times more light to saturate [24, 25]. Although it is now well established that rods can continue to provide some visual information under high light levels (given sufficient contrast and time to light adapt [41, 47, 49]), this higher range of light intensities corresponds to that where cones and melanopsin are expected to provide the primary source of information. Consistent with that view, under the photopic conditions used in our study, we found that relatively large contrasts for rods (in the absence or presence of simultaneous cone contrast; e.g. Figs. 3, 5 and 7) were not associated with any noticeable modulation of PON neuronal responses. By contrast, we observed very robust cone-based responses across the majority of MR units in the PON. Here then, we were particularly interested in understanding the contribution of these cone-based responses to pupil control under the dynamic changes in illumination a mouse might encounter during exploratory activity around dawn and dusk.

Somewhat surprisingly, given our neurophysiological data and recent reports of cone influences on the human pupil [30–32], we found no evidence of a chromatic component to mouse pupil responses. In principle, there are three possible explanations for this finding, the least interesting of which being that such an effect does exist but that the specific stimuli used were simply not sufficient to reveal it. We think this most unlikely, since we found colour opponent responses in PON neurons were robust across a wide range of different conditions (Figs. 2, 5 and 6 as well as associated Additional files) including in PON recordings that use identical stimuli to those used for pupillography.

A second possibility is that all of the classes of PON MR units we identified contribute to regulation of the pupil and that the chromatic influence is simply removed by averaging across cells with S-ON and S-OFF opponent responses. Although formally possible, again we think this is unlikely. Insofar as the mouse retina clearly contains both ipRGC subtypes that lack colour opponency and others that use specific circuitry to generate such behaviour [15, 28], conceptually it would seem hugely inefficient to signal both sorts of information to the PON (or to use some other mechanism to generate the opponency centrally) only to discard one later in the processing pathway. Moreover, even if this were the case, in the absence of some non-linear contribution of the more numerous S-ON vs. S-OFF opponent units, one would expect to see a much greater S-opsin bias in mouse pupil responses than demonstrated here.

A final possibility then is that the colour-opponent MR units we identify in the PON do not contribute to pupillary control and, instead, it is the population of MR cells that lack colour-opponent responses that play a central role. Consistent with this view, we show that L- and S-cone signals sum in an approximately linear manner to regulate initial components of dynamic changes in pupil size under light adaption, with melanopsin contributions becoming gradually more important for larger/slower changes in illumination. These properties match very well what one would predict based on the average activity of the achromatic MR units we recorded (e.g. compare Figs. 4 and 5) and align with previous knockout mouse data on the time-course of cone and melanopsin-derived influences on mouse pupil responses [25, 51, 52].

This arrangement is, however, in stark contrast to the circadian system, where chromatic signals influence both SCN neurophysiological activity and whole animal physiological timing [27]. Presumably, this distinction reflects the differing sensory requirements of the circadian clock and the pupil light reflex: whereas information about the spectral composition of daylight provides the circadian clock with an additional index of time of day, the utility of such signals for pupil control is less obvious. Instead, given the importance of the pupil for supporting more conventional aspects of vision, combining cone signals in an additive manner is presumably of value in that it provides broadband sensitivity to dynamic changes in illumination, whenever they occur.

An important remaining question then is: what could be the function of the large number of MR units in the PON region that exhibit colour-opponent responses? Existing data suggest that cells in the PON shell rather than core are primarily responsible for pupil control [63]. The small size of these structures, coupled with the resolution with which we can determine unit location, preclude any detailed assessment regarding the anatomical arrangement of chromatic MR units. This observation does, however, support the view that not all PON neurons are involved in pupillary control. Primarily based on anatomical data, the PON has additionally been implicated in oculomotor functions including light-induced blink and rapid eye movements as well as in circadian control [46, 64]. At present, we can only speculate as to which of these possible roles chromatic MR neurons contribute. Nonetheless, the widespread appearance of chromatic responses at the level of the PON strongly support previous suggestions of an important role for colour signals in NIF visual processing [33], potentially extending beyond circadian photoentrainment.

### Species differences in pupil control

Our data regarding the timescales over which melanopsin contributions to PON activity/pupil control emerge align well with previous data from macaque and human [30, 65], suggesting a well-conserved contribution of this photoreceptor channel. By contrast, our finding that chromatic signals do not noticeably contribute to mouse pupil regulation suggests a clear distinction from humans, where S- (and potentially also M-) cones contribute opponent signals to those arising from the other opsins [30–32, 66].

Insofar as mice are clearly less specialised towards chromatic photopic vision compared to humans, such a divergence might simply be written off as an expected consequence of the differences between mouse and human retina. Nonetheless, mice retain the S-cone-specific circuitry required to generate a chromatic channel equivalent to human blue-yellow colour vision [67], in addition to mechanisms of colour opponency analogous to those proposed to underlie primate red-green colour discrimination [59]. As such, mice can use colour both as a circadian timing cue [27] and for discrimination at the behavioural level [68], including adapting to use information from novel photopigments based on existing retinal architecture [69]. Most significantly though, we directly demonstrate here that colour information is readily apparent at the level of mouse PON neurons just as it is presumed to be in humans [29].

Given the points raised above, if including colour information in pupil regulation were of value to mice, we see no reason why this would not be apparent. As such, we assume the divergence in the contribution of cones to mouse vs. human pupil control instead reflects the distinct sensory requirements of these two species. While the value of including a chromatic component in human pupil regulation is currently unclear, in the case of mice, we see clear value in using short- and longer-wavelength-sensitive cones in an additive manner. Indeed, as discussed above, this enables rapid pupil constriction to light increments across the visible spectrum, an arrangement that is perhaps more important for preventing photoreceptor saturation in the rod dominated retina in this species vs. the cone-dominant retina of humans.

## Conclusion

Our data provide new insight into the sensory control of PON neuronal activity and the mouse pupil light reflex. The widespread co-occurrence of melanopsin and chromatic signals implies an important role for colour in non-image-forming visual responses, while the lack of chromatic influences on pupillary relative to circadian control demonstrates how distinct ipRGC-dependent physiological responses in mice are individually tuned based on their unique sensory requirements.

## Methods

### Animals

All experiments were conducted in accordance with the Animals (Scientific Procedures) Act of 1986 (United Kingdom) and received approval from the institutional ethics committee at the University of Manchester. Mice were bred and housed at the University of Manchester in a 12:12-h light dark cycle at 22 °C with food and water available ad libitum. Most experiments were performed in adult male red cone knockin (*Opn1mw*^*R*^) mice [34]. For a subset of experiments, we used hemi/homozygous offspring of a red cone knockin/melanopsin knockout [5] cross (*Opn1mw*^*R*^*; Opn4*^*−/−*^). A further subset employed mice lacking the cone-specific cyclic nucleotide-gated channel (*Cnga3*^*−/−*^; [70]).

### In vivo electrophysiology

Animals were anaesthetised with urethane (1.55 g/kg i.p.; Sigma-Aldrich, Dorset, UK) and prepared for stereotaxic surgery as described previously [71]. In brief, a small craniotomy (< 1 mm diameter) was placed 1.1 mm lateral and 2.7 mm posterior to the bregma (based on the coordinates of the OPN from the stereotaxic mouse atlas [72]), and atropine (1% solution in saline) followed by mineral oil (both from Sigma-Aldrich) was applied to the eyes to achieve pupil dilation and to prevent corneal drying without modulating optical transmission. Silicon multielectrode recording probes (32 channel) were then coated in CM-DiI (V22888; Fisher Scientific, Loughborough, UK) to allow post hoc visualisation in histological images, before being inserted into the brain using a hydraulic micromanipulator (MO-10, Narishige International Ltd., London, UK) at 10° to the dorsal-ventral axis and to a depth of 2.3 mm from the pial surface. Experiments employed a variety of silicon recording array designs; either 4 × 8 probes (*n* = 14 experiments; 200 × 50 μm spacing; A4x8-5mm-50-200-177; Neuronexus Technologies Inc., USA), 4 × 2 tetrode arrays (*n* = 19; 200 × 150 μm inter-tetrode spacing, 25 μm intra-tetrode spacing; A4x2tet-5mm-150-200-121; Neuronexus) or 2 × 16 parallel arrays (*n* = 2; 250 × 20 μm spacing; Cambridge NeuroTech, Cambridge, UK). In all cases, prior to neurophysiological recording, mice were left for 30 min to dark to adapt and allow neural activity to stabilise.

Neuronal data was acquired using a Recorder64 data acquisition system (Plexon, USA), amplified for a gain of 3500×, digitised at 40 kHz and stored continuously in a 16-bit format. Single unit activity was isolated using an automated template-matching-based algorithm (Kilosort; [73]) and manually refined using Phy [74]. For initial experiments (*n* = 8), we also extracted virtual tetrode waveforms and sorted manually in Offline Sorter (Plexon, TX, USA), as described previously [71]. Single unit isolation was confirmed by reference to MANOVA F statistics, J3 and Davies-Bouldin validity metrics and the presence of a distinct refractory period (> 1.5 ms) in the interspike interval distribution. Unit isolation resulting from the semi-automated procedure was essentially identical to that derived manually.

### Visual stimuli and modelling

All light measurements were performed using a calibrated spectroradiometer (Bentham instruments, Reading, UK) and quantified according to the known opsin sensitivities after correction for prereceptoral filtering [75, 76] as described previously [27, 54]. For modelling (Fig. 3), we adjusted S- and L-cone opsin sensitivity by ± 25 nm relative to λmax used for calculations below (S-opsin: 365 nm, L-opsin: 556 nm). For this analysis, we used a two-phase saturating function that approximated published measurement mouse lens sensitivity [76] so that we could assess the impact of varying shortwave cutoff (again by ± 25 nm relative to a reported value of 337 nm). Penumbral cone sensitivity was estimated using the method previously presented [50].

Visual stimuli were created using a bespoke light source (components from Thorlabs: NJ, USA, and Edmund Optics; York, UK) which combined signals from three LEDs (λmax 410, 470 and 617 nm; see Fig. 1) via dichroic mirrors. Light was supplied to the subject via a 7-mm diameter flexible fibre optic light guide positioned 5 mm from the contralateral eye and enclosed within an internally reflective plastic cone to provide approximately full field illumination and prevent any light reaching the ipsilateral eye. A similar apparatus was positioned over the ipsilateral eye, providing light (where required) from a single 410-nm LED. LED intensity was controlled dynamically via a PC running LabVIEW and a USB-6343 DAQ board (National Instruments, TX, USA). Further control was provided by neutral density (ND) filter wheels, allowing for spectrally neutral 10 to 10,000-fold (ND1-ND4) reductions in light intensity.

For identification of cells that received input from ipRGCs, we compared responses to two stimuli: a simple monochromatic 460-nm light step (‘Mel High’) and a combination of 410 and 617 nm light (‘Mel Low’; Fig. 1a). Stimuli were presented in interleaved fashion as 10-s steps from a background of darkness (60 s ISI; 10 repeats/stimulus at ND2–0). Intensities of the two stimuli were calibrated to provide identical activation of mouse S- and L-(*Opn1mw*^*R*^) cone opsins (12.8 and 15.5 Log effective photons/cm^2^/s respectively at ND0) but to differ greatly in their ability to activate melanopsin (16.0 vs. 13.4 Log effective photons/cm^2^/s for Mel High and Low). Although these stimuli, in principle, also differ substantially in apparent brightness for rods, effective rod flux for both stimuli was expected to be sufficiently high by ND1 (12.9 Log effective photons/cm^2^/s for Mel Low) to evoke a maximal rod response [42, 77, 78].

For selective manipulation of cone photoreception, we first calibrated a mixture of each LED to approximate a wildtype mouse’s experience of natural daylight (Fig. 2a, b), based on previously reported data [27]. Input-output relationships for modulations in LED intensity relative to this background were measured and linearized by 4th order polynomial fits. We then calculated, as above, modulations in the intensity of each LED that provided changes in the activation of L- and/or S- cone opsins (in isolation, unison or antiphase) by ± 75% relative to the background (equivalent to a 0.85 log unit or sevenfold change in apparent brightness for the stimulated opsin). For L- and S-cone-isolating stimuli, effective change in brightness for the silenced opsin was calculated to be < 0.2% Michelson and for all stimuli effective changes in rod and melanopsin activation were below 6% (full details of all stimuli are provided in Additional file 12). We also produced stimuli providing spectrally neutral modulations in light intensity (± 75% relative to the background in Fig. 1c) that allowed for concurrent modulations in the activation of all opsins and stimuli designed to provide a large spectral change but to be silent for cones (Fig. 3a; < 0.05% Michelson contrast for cones, 45.0 and 43.2% respectively for rods and melanopsin).

For assessment of contrast response relationships, we applied cone-isolating and all-opsin stimuli as 0.25-Hz square-wave modulations providing 15–75% Michelson contrast (albeit with a smooth 40-ms transition between ‘bright’ and ‘dim’ phases). Opsin-specific stimuli were presented as interleaved blocks of 6 cycles running from low to high contrast to control for any effect of contrast adaptation. The full protocol was then repeated five times to provide 30 repeats for each stimulus. Stimuli were presented well within the photopic regime (as shown in Fig. 2b) to allow for assessment of melanopsin-cone interactions (comparison of L + S and all opsin stimuli) without rod intrusion [40]. In a subset of experiments, we first also applied the same protocol at ND1, to confirm cone-based responses were stable across the photopic range. For experiments assessing temporal frequency tuning, cone-isolating and all-opsin stimuli were applied exclusively at ND0 as sinusoidal modulations (0.025–5 Hz) at a fixed contrast of 75% Michelson (interleaved as above although here the number of stimulus cycles ranged from 10 at 0.025 Hz to 80 at 5 Hz).

For all stimuli involving cone-isolating stimuli, background light intensity at the ipsilateral eye was set to approximate that for the stimulated eye using a 410-nm LED (which provides near equal activation of all mouse opsin classes; [71]). The one exception here was for an additional protocol applied in a subset of experiments (*n* = 5 *Opn1mw*^*R*^ mice, *n* = 4 *Opn1mw*^*R*^;*Opn4*^*−/−*^) where cone-isolating stimuli were applied using identical parameters to those used for pupillography (below), interleaved single cycle square-wave modulations (75% contrast, 5 s/phase, positive first and negative first polarity).

### Histology

After each experiment, brains were removed and placed into 4% paraformaldehyde for 48 h before cryoprotection in 30% sucrose (24 h). Brains were then frozen with dry ice and sectioned coronally using a freezing sledge microtome at widths of 100 μm before mounting with Vectashield (Vector laboratories, UK) to glass slides and coverslipping. Sections were imaged under an upright light microscope (BX51; Olympus, UK) with appropriate filter sets for visualisation of DiI fluorescence and images acquired with a Coolsnap HQ camera (Photometrics, USA). Resulting images were scaled and aligned with best matching coronal panels from the mouse atlas [72] with the anatomical location for each cell estimated based on the known geometry of the recording array and the corresponding recording site location were largest spike amplitudes were detected. For display (Additional file 3: Figure S3), estimated unit locations were mapped onto a single coronal anatomical template, based on the projected centre of the PON from each recording.

### Pupillography

Animals were habituated prior to handling and had their whiskers trimmed prior to experiments to reduce interference in the images. All experiments were performed during the middle of the animal’s light phase and during experiments the room was maintained in constant darkness. For assessment of consensual pupil responses, the left pupil was dilated by topical application of tropicamide (1% solution; Minimus, UK), mice were gently restrained and the same light source used for neurophysiological recording placed over the dilated pupil. A NoIR Raspberry Pi camera (V2.1; Raspberry Pi Foundation, UK) with a macro lens and visible light filter (695 nm longpass; Thorlabs, USA) was then placed at a fixed distance (5 cm) from the right eye and an 880-nm infrared light (Thorlabs, NJ, USA) was then placed above to enhance pupil-iris contrast. Images were acquired at 2 Hz, synchronised to visual stimulus presentation via LabVIEW.

Mice were exposed to the background stimulus for 30 s prior to application of cone-isolating and all photoreceptor stimuli (as above) as single cycle square-wave modulations (75% contrast, 5 s/phase) at ND1. Stimuli were presented to each animal in randomised order and at both possible polarities (i.e. transition from background to ‘bright’ followed by ‘dim’ and vice versa) with individual stimuli separated by 10 s of exposure to the background. Each animal received two to four trials of each stimulus/polarity (mean = 3.1 trials/stimulus).

For analysis, images (2592 × 1944 pixels) were virtually stacked and stabilised using After Effects (Adobe Software Systems Ltd., Ireland) then cropped to the horizontal and vertical boundaries of the eye. The images were then processed using custom MATLAB scripts (Mathworks, USA), based on published methodology [79], to extract pupil diameter. In brief, images were first converted to greyscale and a 3 × 3 median filter applied to reduce noise. A region-growing algorithm was run from an initial, user-defined, seed point. The resulting binary image then had any holes generated by reflections filled before undergoing a morphological opening operation with a circular 5 × 5 structuring element for smoothing. The image boundary was subsequently extracted by subtraction from a morphologically dilated representation (using a 3 × 3 structuring element) and an ellipse fitted using the direct fit method [80]. Pupil diameter (in pixels) was taken as the long axis of the fitted ellipse and normalised according to eye size (long axis of an ellipse fitted to the whole eye).

### Data analysis

For initial cell classification, units were considered to exhibit sustained responses when the change in firing evoked by the Mel High stimulus remained above 25% of its initial value (mean over the first 500 ms) over the last 5 s of the 10-s light step (and did not fall below 1.2 spikes/s above pre-step values). Cells that otherwise exhibited significant increases in firing at the onset of the light step were designated ‘transient’ and cells that exhibited significant decreases as ‘OFF’. We further separated cells with sustained responses based on the presence of equivalent initial responses to Mel High vs. Low (100 ms bin occurring within the first 500 ms of light onset with the highest firing rate) at the two higher intensities (indicating equivalent rod/cone-driven components of the response). For subsequent analysis, we then extracted the mean change in the firing rate for each cell (relative to the preceding 10-s baseline) across early (first 500 ms) or late (last 5 s) elements of the response and analysed with two-way RM ANOVA (GraphPad Prism 7; GraphPad Software, Inc., CA, USA).

For analysis of neuronal responses to cone-isolating stimuli, spike counts were binned (100 bins/stimulus cycle; smoothed with a 5-bin boxcar filter) and peak-trough amplitudes extracted. To remove the effect of random variations in baseline firing, we also produced similar estimates based on shuffled data (spike counts shuffled in time independently for each trial). Cells were considered responsive when the measured response amplitude exceed the 95% confidence limits of responses assessed from shuffled data (100 repeats), with the mean shuffled response subsequently subtracted from the true response. Response polarity (ON vs. OFF) was assessed based on the stimulus phase where we observed the largest absolute deviation in spike rates from the mean and the sign (positive vs. negative) of that response. To determine cone opsin preference, the mean response across all tested contrasts was determined across each of the four cardinal stimulus dimensions, normalised according to the largest absolute magnitude and represented as vectors with ON and OFF responses arbitrarily designated as vectors at 0–180°, 45–225°, 90–270° and 135–315° for L, L + S, S and L − S stimuli respectively. Overall preference was then taken as the mean of the resulting vectors. Cells were categorised as colour opponent when we observed significant responses (as above) of opposite sign for L- and S-opsin-isolating stimuli or when we did not detect a significant response from one of the two cone opsins in isolation but found that the mean response to the L − S stimuli was significantly greater than that to the L + S stimulus (*t* test, *P* < 0.05).

For comparisons of contrast response functions and temporal frequency tuning we also used a modification of the *χ*^2^-peridogram [27, 81] to quantify the percentage variance within the timeseries that was accounted for by a rhythmic process with the same periodicity as the stimulus. This provides an unbiased assessment of relative response strength that has the advantages that it makes no assumptions about the shape of neuronal responses, allows for direct significance testing and allows for comparison of responses occurring across greatly differencing timescales. As above, for group comparison of responses between stimuli, we used two-way RM ANOVA.

For quantification of pupillary responses, we extracted the mean pupil size across 5-s epochs corresponding to ‘bright’ and ‘dim’ stimulus phases (expressed as a percent change relative to baseline) and compared via paired *t* test. Further analyses were performed on the raw difference between bright and dim stimulus phases (normalised to eye size) using two-way and one-way RM ANOVA as indicated in the manuscript text.

Throughout the manuscript, appropriate post hoc tests were employed wherever main ANOVA reported a significant effect of stimulus or interaction and significant differences (*P* < 0.05) between stimuli indicated wherever they occurred. Full details of all statistical test results and the underlying raw data is provided in the relevant worksheets of the accompanying data (Additional files 12 and 13).

## Additional files

Additional file 1: **Figure S1.** Cells that lack sustained excitation exhibit equivalent responses to light steps providing divergent melanopsin excitation. (**a**, **c**, **e**, **g**) Mean ± SEM normalised change in firing for Mel High and Low steps across 3 logarithmically spaced intensities for *Opn1mw*^*R*^ transient (**a**; *n* = 121) or OFF cells (**c**; *n* = 37), *Opn1mw*^*R*^*; Opn4*^*−/−*^ transient (**f**; *n* = 24) and *Cnga3*^*−/−*^ transient cells (**g**; *n* = 15). Shaded regions represent epochs of darkness. No OFF cells were identified in *Opn1mw*^*R*^*; Opn4*^*−/−*^ and only one cell found in *Cnga3*^*−/−*^. (**b**, **d**, **f**, **h**) Mean ± SEM change in firing observed during first 500 ms of the Mel High and Low light steps for corresponding cell populations in **a**, **c**, **f** and **g**. Data were analysed by two-way RM ANOVA, with Sidak’s post-tests at each intensity when significant main effects of stimulus or StimulusxIrradiance were identified. ** = *P* < 0.01, otherwise *P* > 0.05. (JPG 311 kb)

Additional file 2: **Figure S2.** An unexpected influence of rods at high irradiance in a small subset of sustained cells. (**a**, **c**) Mean ± SEM normalised change in firing for Mel High and Low steps across 3 logarithmically spaced intensities for *Opn1mw*^*R*^ (**a**; *n =* 12) and *Opn1mw*^*R*^*; Opn4*^*−/−*^ (**c**; *n =* 8) sustained cells with globally enhanced responses to Mel High stimuli at high irradiance. Shaded regions represent epochs of darkness. (**b**, **d**) Mean ± SEM change in firing observed during first 500 ms of the Mel High and Low light steps for corresponding cell populations in **a** and **c**. Data were analysed by two-way RM ANOVA with Sidak’s post-tests at each intensity when significant main effects of stimulus or StimulusxIrradiance were identified. * and *** = *P* < 0.05 and 0.001, otherwise *P >* 0.05. (**e, f**) Example responses for 1 (of three) *Cnga3*^*−/−*^ cells (**e**) and early/late response quantification for all three cells (**f**) with analogous response properties. Conventions otherwise as in **a**-**d.** (JPG 291 kb)

Additional file 3: **Figure S3.** Relationship between anatomical location and visual response properties for mouse pretectal neurons. (**a**) Left: Histological image of DiI marked probe tracks (red) and light microscopic image (pseudocoloured green), Right: schematic of probe sites aligned with corresponding stereotaxic atlas figure. (**b**) Anatomical locations of MR (left) and non-MR units (right) with varying responses to cone-isolating stimuli, aligned according to probe position relative to projected PON centre for each experiment (se methods). (JPG 152 kb)

Additional file 4: **Figure S4.** Cone inputs to non-melanopsin-responsive pretectal neurons. (**a, c, e**) Left: Examples of transient (**a**), OFF (**c**) and sustained (**e**) non-MR units responses to 75% contrast cone-isolating stimuli. Right: opsin preference plots for each unit, conventions as in Fig. 2. (**b, d, f**) Mean ± SEM baseline subtracted, normalised, responses for main subpopulations of transient (**b**), OFF (**d**), and sustained (**f**) non-MR units to cone-isolating stimuli (n numbers for each group shown indicated in **g**). (**g**) Proportions of non-MR units exhibiting each response type; significant differences from MR units determined by Fisher’s exact test. (**h, i**) Mean ± SEM contrast response relationships of opponent (**e**; *n* = 25) or non-opponent (**f**, *n* = 64) MR cells for single opsin stimuli (left) or for stimuli modulating both cone opsins in unison or antiphase (right). Conventions and analysis (two-way RM ANOVA with Sidak’s post-test) as in Fig. 2. *,** and *** represent *P* < 0.05, *P* < 0.01 and *P* < 0.001. (JPG 428 kb) (JPG 427 kb)

Additional file 5: **Figure S5.** Cone opsin preference is maintained at reduced irradiance. (**a**) Scatter plot showing opsin preferences for all units tested at both ND0 and ND1 (*n =* 37 MR and *n* = 96 non-MR units). Note the strong correlation between response properties under both conditions (fit to y = x, *r* = 0.99). A small subset of cells only exhibited robust responses at one of the two irradiances (ND1-only *n =* 3/37MR and 9/96 non MR; ND0-only 6/96 non-MR units; n.r. = no detectable response). (**b**) Scatter plot sowing maximal response to cone-isolating stimuli at ND0 and ND1 for all cells with robust responses under at least one condition (top; *n* = 34 MR units and 60 non-MR units) and frequency distribution showing difference in maximal response amplitude at ND1-ND0 (bottom). *** indicates *P <* 0.001 from paired *t* test between response at ND1 and ND0. (**c**) Mean ± SEM responses (normalised to max for each cell under any condition) to 75% contrast cone-isolating stimuli at ND1 and ND0 for non-opponent (top, *n* = 14), S-ON/L-OFF (middle, *n* = 16) and L-ON/S-OFF (bottom; *n* = 4) MR units that responded under either intensity. (JPG 177 kb)

Additional file 6: **Figure S6.** Cone inputs to pretectal neurons in melanopsin knockout mice. (**a**) Left: Examples of non-opponent, S-ON/L-OFF and L-ON/S-OFF units in *Opn1mw*^*R*^*;Opn4*^*−/−*^mice tested with 75% contrast cone-isolating stimuli at ND0. Right: opsin preference plots for each unit, conventions as in Fig. 2. (**b**) Mean ± SEM baseline subtracted, normalised, responses for all non-opponent and S-ON/L-OFF prectectal *Opn1mw*^*R*^*;Opn4*^*−/−*^ units (n numbers for each group shown indicated in **c**). (**c**) Left: opsin preference plots for all responsive *Opn1mw*^*R*^*;Opn4*^*−/−*^ units; Middle: Proportions of visually responsive *Opn1mw*^*R*^*;Opn4*^*−/−*^ units exhibiting each cone-response type (from 5 mice); χ^2^-test indicated this distribution was statistically equivalent to that observed in *Opn1mw*^*R*^ cells (Right). (**d, e**) Mean ± SEM contrast response relationships of opponent (**d**; *n* = 10) or non-opponent (**e**, *n* = 13) *Opn1mw*^*R*^*;Opn4*^*−/−*^ cells for single opsin stimuli (left) or for stimuli modulating both cone opsins (right) at ND0. Conventions and analysis (two-way RM ANOVA with Sidak’s post-test) as in Fig. 2. *,** and *** represent *P <* 0.05, *P <* 0.01 and *P* < 0.001. (**f**) Scatter plot showing opsin preferences for *Opn1mw*^*R*^*;Opn4*^*−/−*^ cells tested at both ND0 and ND1 (*n* = 34 of 41 cells contributing to panels above from 4 of the 5 mice tested at ND0; conventions as in Additional file 5: Figure S5a), revealing a strong correlation between response properties under both conditions (fit to y = x, *r* = 0.95). Few cells exhibited robust responses at only one of the two irradiances (ND1-only *n* = 7/34; ND0-only *n =* 1/34). (**g**) Scatter plot sowing maximal response to cone-isolating stimuli at ND0 and ND1 for *Opn1mw*^*R*^*;Opn4*^*−/−*^ cells with robust responses under at least one condition (*n* = 25 units) and frequency distribution showing difference in maximal response amplitude at ND1-ND0 (bottom). ** indicates *P <* 0.01 from paired *t* test between response at ND1 and ND0. (JPG 368 kb)

Additional file 7: **Figure S7.** Additional validation of cone-isolating stimuli. (**a**) Mean ± SEM responses of colour opponent and non-opponent MR and non-MR units in *Opn1mw*^*R*^ and *Opn1mw*^*R*^*;Opn4*^*−/−*^mice that responded at ND1 *(*conventions as in Fig. 3b). Data were analysed by one-way RM ANOVA with Dunnett’s post-test (clockwise from top left *n* = 19, 18, 7, 17, 36 and 15). (**c**) Top: Mean ± SEM responses of *Cnga3*^*−/−*^, *Opn1mw*^*R*^ and *Opn1mw*^*R*^*;Opn4*^*−/−*^ neurons to 75% contrast cone-isolating stimuli; (analysis includes all light-responsive cells tested in all genotypes; *n* = 24, *n* = 230 and *n* = 41 respectively). Bottom: cumulative frequency distribution for maximal response evoked by cone-isolating stimuli in the same populations of cells. Data were analysed by Kruskal-Wallis test with Dunn’s test for multiple comparisons. *,**,*** indicate *P <* 0.05, *P <* 0.01 and *P <* 0.001 respectively; ns indicates *P* > 0.05. (**c**) Changes in cone-opsin contrast for cone-silent stimuli as a result of varying both λmax and contribution of penumbral cones (conventions as in Fig. 3f, i). (JPG 135 kb)

Additional file 8: **Figure S8.** Opponent and non-opponent responses of pretectal neurons from *Opn1mw*^*R*^*;Opn4*^*−/−*^ mice under the stimulus paradigm used for pupillography. (**a**, **b**) Mean ± SEM baseline subtracted responses of S-ON/L-OFF (**a**; *n =* 6) and non-opponent (**b**; *n* = 18) *Opn1mw*^*R*^*;Opn4*^*−/−*^ units (from 4 mice) to 75% contrast cone isolating and all opsin stimuli delivered as for pupillography (Fig. 5). (**c**, **d**) Left: Mean ± SEM change in firing between ‘dim’ and ‘bright’ stimulus phases for all stimuli (averaged across full 5 s phase and both stimulus polarities) for S-ON/L-OFF (**c**) and non-opponent units (**d**) as above. Right: Mean ± SEM change in firing between ‘dim’ and ‘bright’ stimulus phases for 75% contrast stimuli targeting L + S cone opsins or all photoreceptors as a function of time since contrast step (averaged across both stimulus polarities as above). Data analysed by two-way RM ANOVA with Sidak’s post-tests. Non-opponent units lacked any stimulus-related differences, but a nominally significant increase in S-ON/L-OFF neuronal responses at 3 s but not earlier or later timepoints was observed (*P* = 0.03). (JPG 294 kb)

Additional file 9: **Figure S9.** Temporal tuning of cone inputs to non-melanopsin-responsive cells. (**a-c**) Example peristimulus firing rate histograms for transient (**a**), OFF (**b**) and sustained (**c**) non-MR neurons in *Opn1mw*^*R*^ mice, tested with sinusoidal oscillations of their optimal cone stimulus type (L − S modulation for chromatic units and L + S stimulus for the non-opponent units – rightmost traces in each panel) at 75% contrast and varying temporal frequency. (JPG 275 kb)

Additional file 10: **Figure S10.** Non-melanopsin-responsive neurons display equivalent responses to stimuli activating both cone opsins in the presence or absence of contrast for other photoreceptors. (**a**, **c, e**) Mean ± SEM responses of *Opn1mw*^*R*^ transient units (**a**; *n =* 62) and OFF (**c**; *n =* 16) and sustained (**e**; *n* = 11) cells to rapid (0.25 Hz square wave; top) or gradual (0.025 Hz sinusoid; botom) spectrally neutral stimulus modulations (all opsins) and stimuli targeting just L- and S-cone opsin (75% contrast). (**b, d, f**) Contrast (top) and temporal frequency (bottom) tuning curves for *Opn1mw*^*R*^ transient (**b**), OFF (**d**) and sustained (**f**) responses to all opsin and L + S-opsin-isolating stimuli (as above). For contrast response analysis, data points represent difference in mean firing rate during the last 400 ms at ‘bright’ vs. ‘dim’ stimulus phases. For temporal frequency analysis data points represent the % variance in firing rate accounted for the stimulus. In both cases data analysed by two-way RM ANOVA with Sidak’s post-tests. *** = *P* < 0.001. (JPG 269 kb)

Additional file 11: **Figure S11.** Responses to cone-selective and all-opsin contrast at lower irradiance. (**a**) Mean ± SEM responses of *Opn1mw*^*R*^ MR (left; *n =* 34) and *Opn1mw*^*R*^*;Opn4*−/− (right; *n =* 25) units tested at ND1 with 60% contrast stimuli modulating L + S opsin or all-opsins. (**b**) Contrast tuning curves for *Opn1mw*^*R*^ MR (left) and *Opn1mw*^*R*^*; Opn4*^*−/−*^ (right) responses to all opsin and L + S-opsin-isolating stimuli (as above). Data points represent difference in mean firing rate during the last 400 ms at ‘bright’ vs. ‘dim’ stimulus phases. Data analysed by two-way RM ANOVA with Sidak’s post-tests. *** = *P <* 0.001. (JPG 113 kb)

Additional file 12: Raw and analysed data (including statistical results) for all electrophysiological recordings, light measurements and pupillographic data used to generate Figs. 1, 2, 3, 4, 5, 6 and 7. (XLSX 6532 kb)

Additional file 13: Raw and analysed data (including statistical results) for all electrophysiological recordings, used to generate Additional files 1, 2, 3, 4, 5, 6, 7, 8, 9, 10 and 11: Figs. S1–11. (XLSX 13404 kb)
