# Supplementary figures and images for: Additive contributions of melanopsin and both cone types provide broadband sensitivity to mouse pupil control
Source: BMC Biol. 2018 Jul 31;16:83. doi: 10.1186/s12915-018-0552-1 (PMC6066930; doi:10.1186/s12915-018-0552-1)

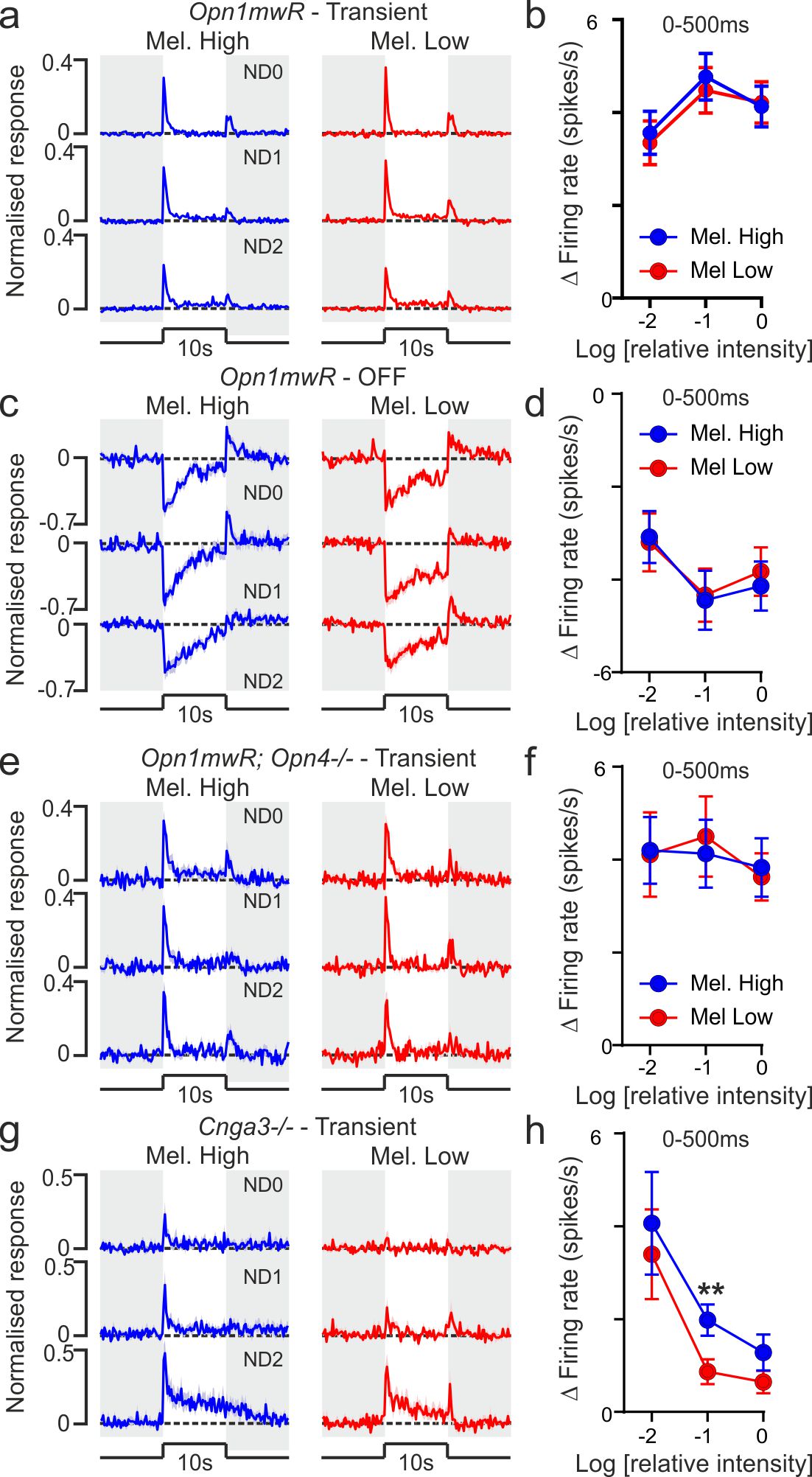

Supplement: Supplementary file 1 — Figure S1. Cells that lack sustained excitation exhibit equivalent responses to light steps providing divergent melanopsin excitation. (a, c, e, g) Mean ± SEM normalised change in firing for Mel High and Low steps across 3 logarithmically spaced intensities for Opn1mwR transient (a; n = 121) or OFF cells (c; n = 37), Opn1mwR; Opn4−/− transient (f; n = 24) and Cnga3−/− transient cells (g; n = 15). Shaded regions represent epochs of darkness. No OFF cells were identified in Opn1mwR; Opn4−/− and only one cell found in Cnga3−/−. (b, d, f, h) Mean ± SEM change in firing observed during first 500 ms of the Mel High and Low light steps for corresponding cell populations in a, c, f and g. Data were analysed by two-way RM ANOVA, with Sidak’s post-tests at each intensity when significant main effects of stimulus or StimulusxIrradiance were identified. ** = P < 0.01, otherwise P > 0.05. (JPG 311 kb) [file 12915_2018_552_MOESM1_ESM.jpg]

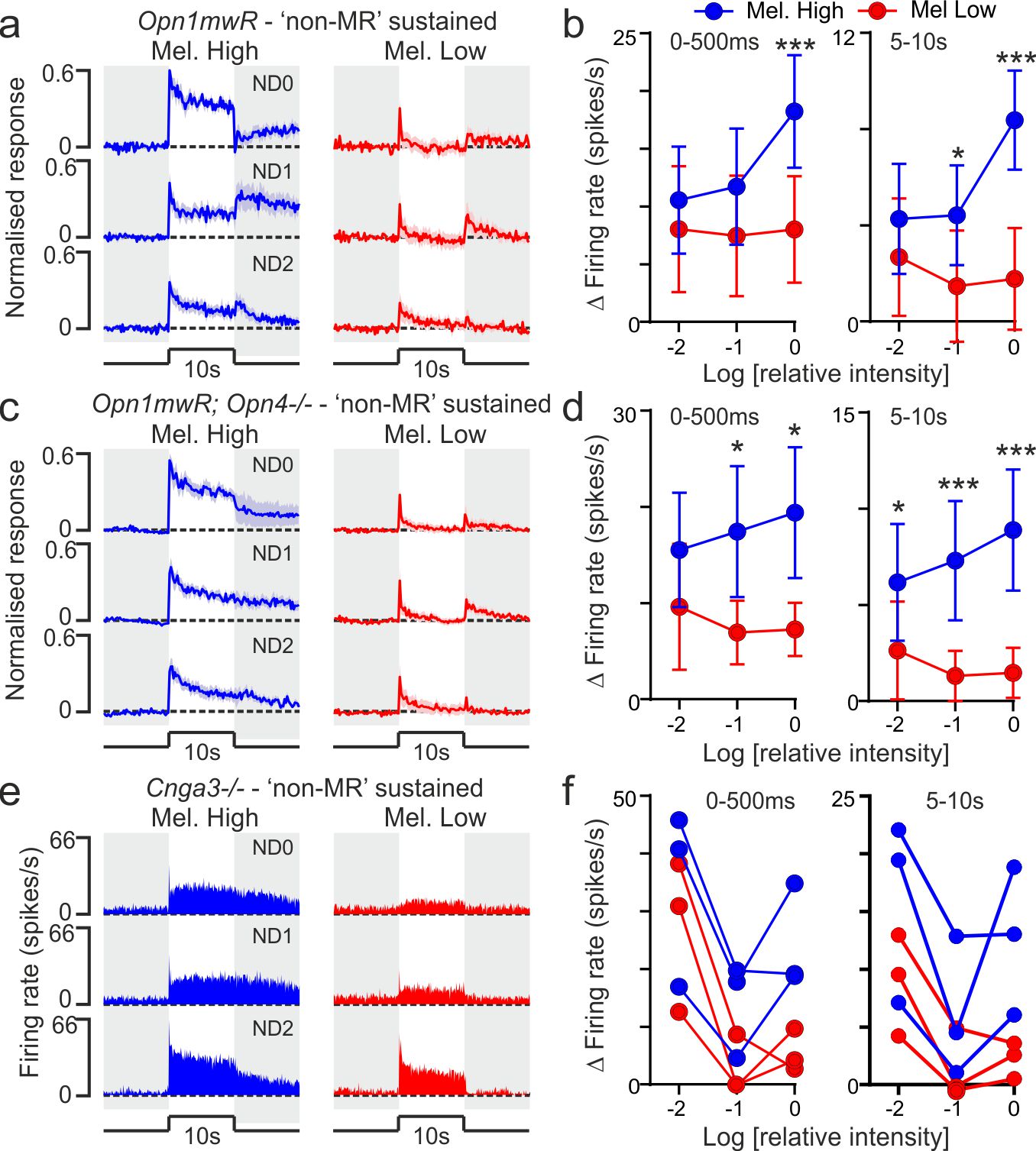

Supplement: Supplementary file 2 — Figure S2. An unexpected influence of rods at high irradiance in a small subset of sustained cells. (a, c) Mean ± SEM normalised change in firing for Mel High and Low steps across 3 logarithmically spaced intensities for Opn1mwR (a; n = 12) and Opn1mwR; Opn4−/− (c; n = 8) sustained cells with globally enhanced responses to Mel High stimuli at high irradiance. Shaded regions represent epochs of darkness. (b, d) Mean ± SEM change in firing observed during first 500 ms of the Mel High and Low light steps for corresponding cell populations in a and c. Data were analysed by two-way RM ANOVA with Sidak’s post-tests at each intensity when significant main effects of stimulus or StimulusxIrradiance were identified. * and *** = P < 0.05 and 0.001, otherwise P > 0.05. (e, f) Example responses for 1 (of three) Cnga3−/− cells (e) and early/late response quantification for all three cells (f) with analogous response properties. Conventions otherwise as in a-d. (JPG 291 kb) [file 12915_2018_552_MOESM2_ESM.jpg]

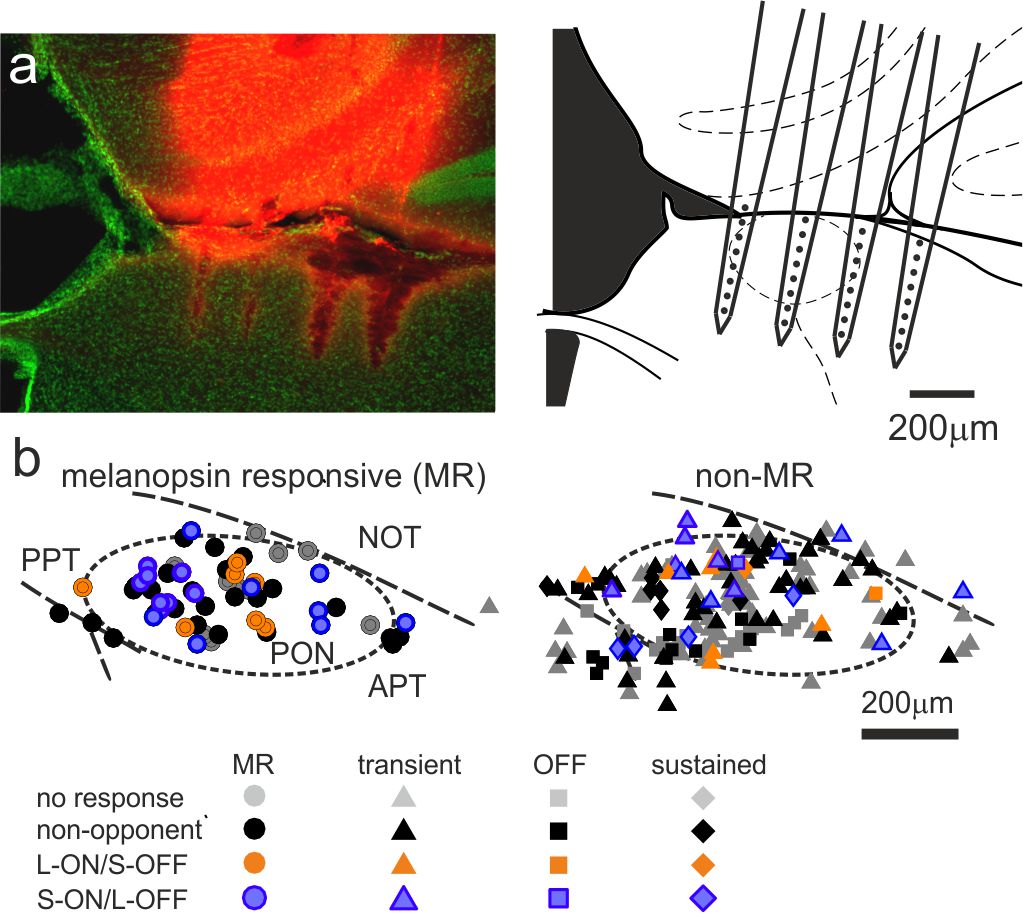

Supplement: Supplementary file 3 — Figure S3. Relationship between anatomical location and visual response properties for mouse pretectal neurons. (a) Left: Histological image of DiI marked probe tracks (red) and light microscopic image (pseudocoloured green), Right: schematic of probe sites aligned with corresponding stereotaxic atlas figure. (b) Anatomical locations of MR (left) and non-MR units (right) with varying responses to cone-isolating stimuli, aligned according to probe position relative to projected PON centre for each experiment (se methods). (JPG 152 kb) [file 12915_2018_552_MOESM3_ESM.jpg]

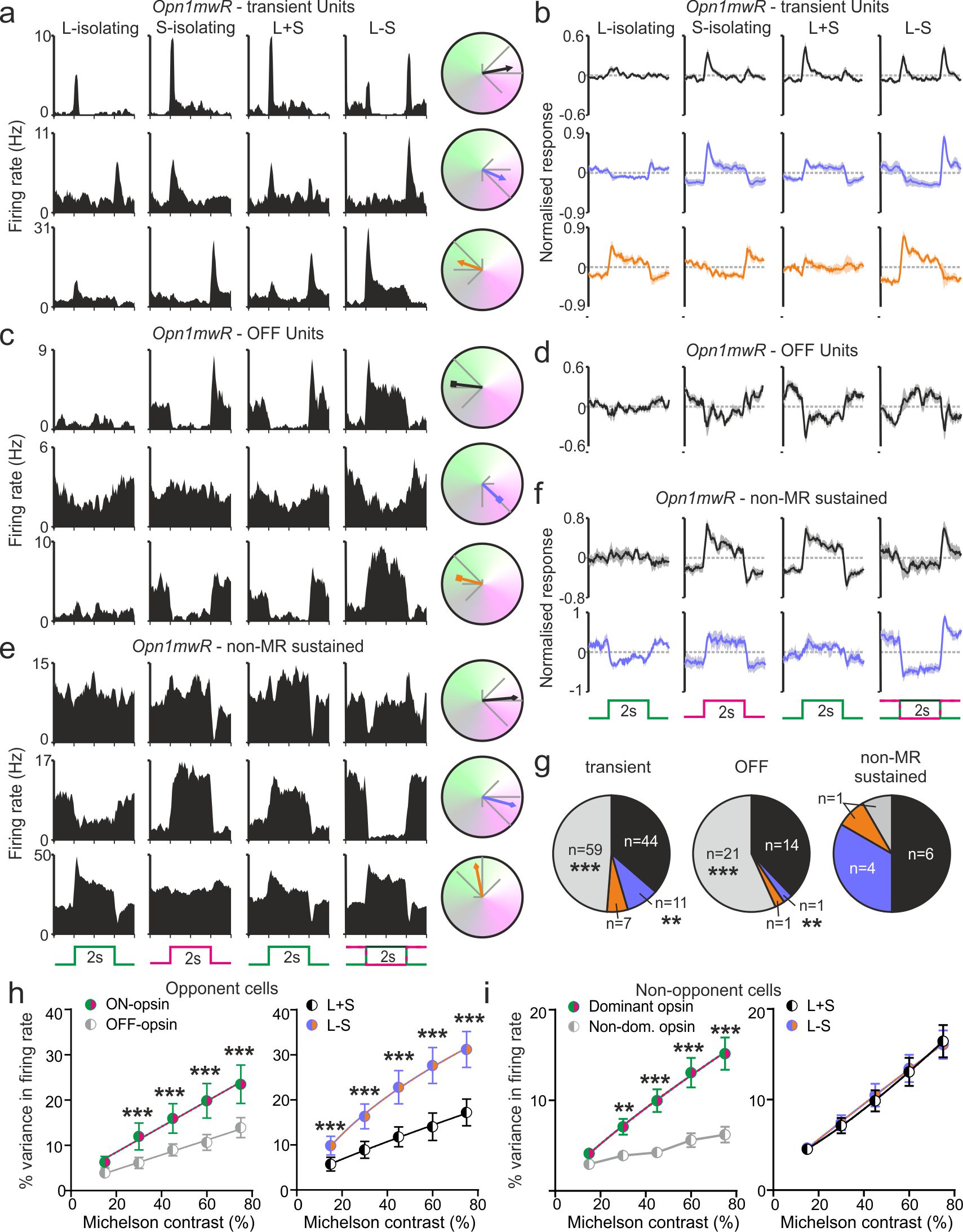

Supplement: Supplementary file 4 — Figure S4. Cone inputs to non-melanopsin-responsive pretectal neurons. (a, c, e) Left: Examples of transient (a), OFF (c) and sustained (e) non-MR units responses to 75% contrast cone-isolating stimuli. Right: opsin preference plots for each unit, conventions as in Fig. 2. (b, d, f) Mean ± SEM baseline subtracted, normalised, responses for main subpopulations of transient (b), OFF (d), and sustained (f) non-MR units to cone-isolating stimuli (n numbers for each group shown indicated in g). (g) Proportions of non-MR units exhibiting each response type; significant differences from MR units determined by Fisher’s exact test. (h, i) Mean ± SEM contrast response relationships of opponent (e; n = 25) or non-opponent (f, n = 64) MR cells for single opsin stimuli (left) or for stimuli modulating both cone opsins in unison or antiphase (right). Conventions and analysis (two-way RM ANOVA with Sidak’s post-test) as in Fig. 2. *,** and *** represent P < 0.05, P < 0.01 and P < 0.001. (JPG 428 kb) (JPG 427 kb) [file 12915_2018_552_MOESM4_ESM.jpg]

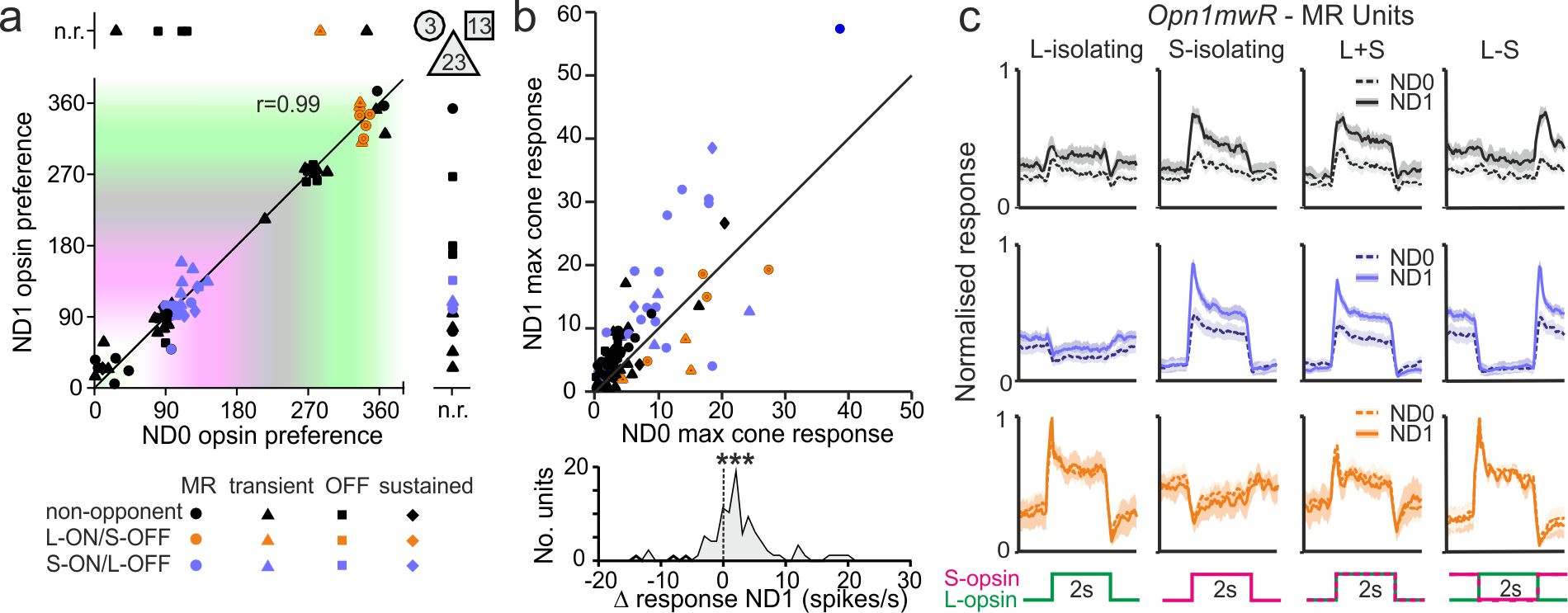

Supplement: Supplementary file 5 — Figure S5. Cone opsin preference is maintained at reduced irradiance. (a) Scatter plot showing opsin preferences for all units tested at both ND0 and ND1 (n = 37 MR and n = 96 non-MR units). Note the strong correlation between response properties under both conditions (fit to y = x, r = 0.99). A small subset of cells only exhibited robust responses at one of the two irradiances (ND1-only n = 3/37MR and 9/96 non MR; ND0-only 6/96 non-MR units; n.r. = no detectable response). (b) Scatter plot sowing maximal response to cone-isolating stimuli at ND0 and ND1 for all cells with robust responses under at least one condition (top; n = 34 MR units and 60 non-MR units) and frequency distribution showing difference in maximal response amplitude at ND1-ND0 (bottom). *** indicates P < 0.001 from paired t test between response at ND1 and ND0. (c) Mean ± SEM responses (normalised to max for each cell under any condition) to 75% contrast cone-isolating stimuli at ND1 and ND0 for non-opponent (top, n = 14), S-ON/L-OFF (middle, n = 16) and L-ON/S-OFF (bottom; n = 4) MR units that responded under either intensity. (JPG 177 kb) [file 12915_2018_552_MOESM5_ESM.jpg]

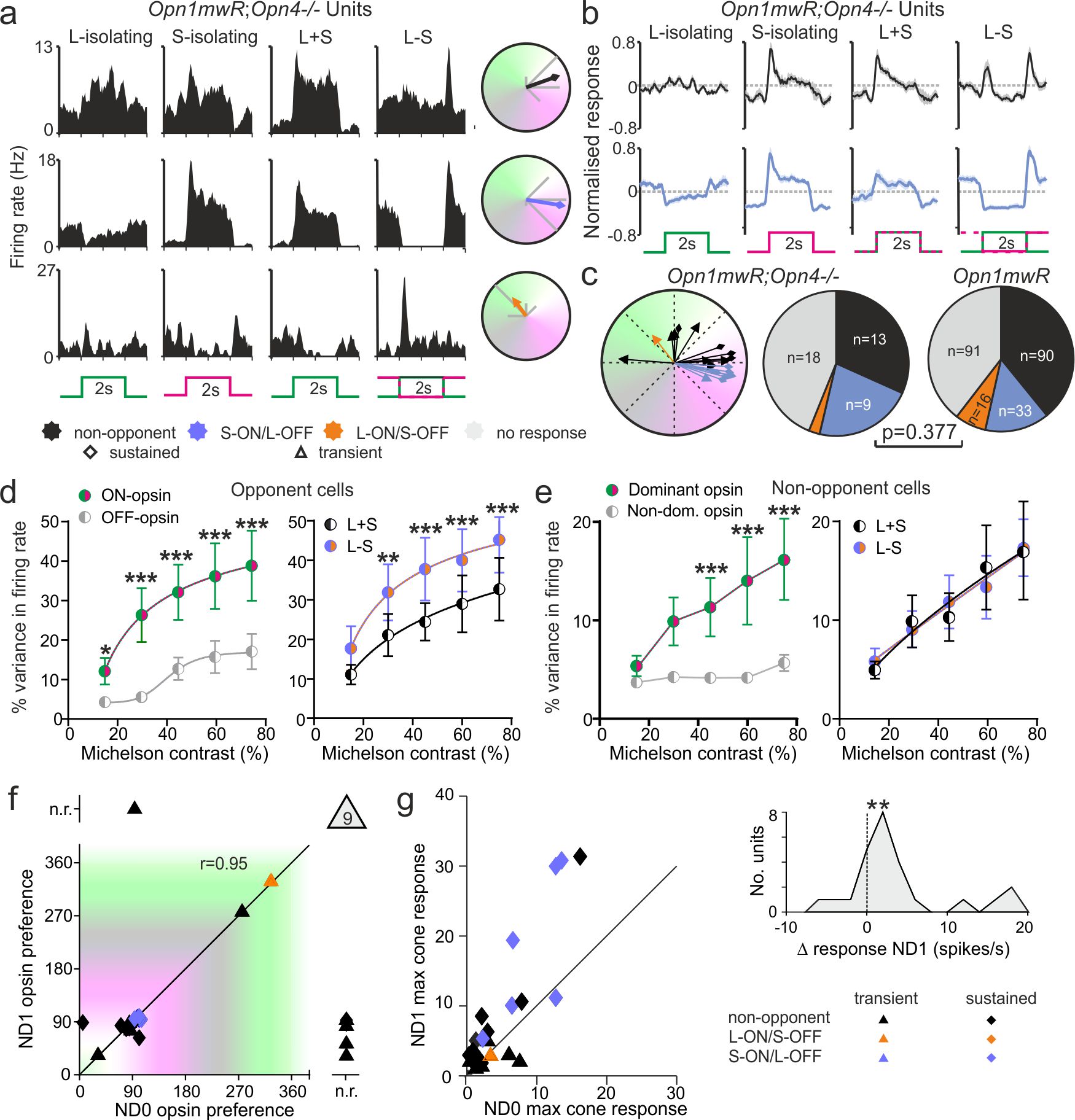

Supplement: Supplementary file 6 — Figure S6. Cone inputs to pretectal neurons in melanopsin knockout mice. (a) Left: Examples of non-opponent, S-ON/L-OFF and L-ON/S-OFF units in Opn1mwR;Opn4−/−mice tested with 75% contrast cone-isolating stimuli at ND0. Right: opsin preference plots for each unit, conventions as in Fig. 2. (b) Mean ± SEM baseline subtracted, normalised, responses for all non-opponent and S-ON/L-OFF prectectal Opn1mwR;Opn4−/− units (n numbers for each group shown indicated in c). (c) Left: opsin preference plots for all responsive Opn1mwR;Opn4−/− units; Middle: Proportions of visually responsive Opn1mwR;Opn4−/− units exhibiting each cone-response type (from 5 mice); χ2-test indicated this distribution was statistically equivalent to that observed in Opn1mwR cells (Right). (d, e) Mean ± SEM contrast response relationships of opponent (d; n = 10) or non-opponent (e, n = 13) Opn1mwR;Opn4−/− cells for single opsin stimuli (left) or for stimuli modulating both cone opsins (right) at ND0. Conventions and analysis (two-way RM ANOVA with Sidak’s post-test) as in Fig. 2. *,** and *** represent P < 0.05, P < 0.01 and P < 0.001. (f) Scatter plot showing opsin preferences for Opn1mwR;Opn4−/− cells tested at both ND0 and ND1 (n = 34 of 41 cells contributing to panels above from 4 of the 5 mice tested at ND0; conventions as in Additional file 5: Figure S5a), revealing a strong correlation between response properties under both conditions (fit to y = x, r = 0.95). Few cells exhibited robust responses at only one of the two irradiances (ND1-only n = 7/34; ND0-only n = 1/34). (g) Scatter plot sowing maximal response to cone-isolating stimuli at ND0 and ND1 for Opn1mwR;Opn4−/− cells with robust responses under at least one condition (n = 25 units) and frequency distribution showing difference in maximal response amplitude at ND1-ND0 (bottom). ** indicates P < 0.01 from paired t test between response at ND1 and ND0. (JPG 368 kb) [file 12915_2018_552_MOESM6_ESM.jpg]

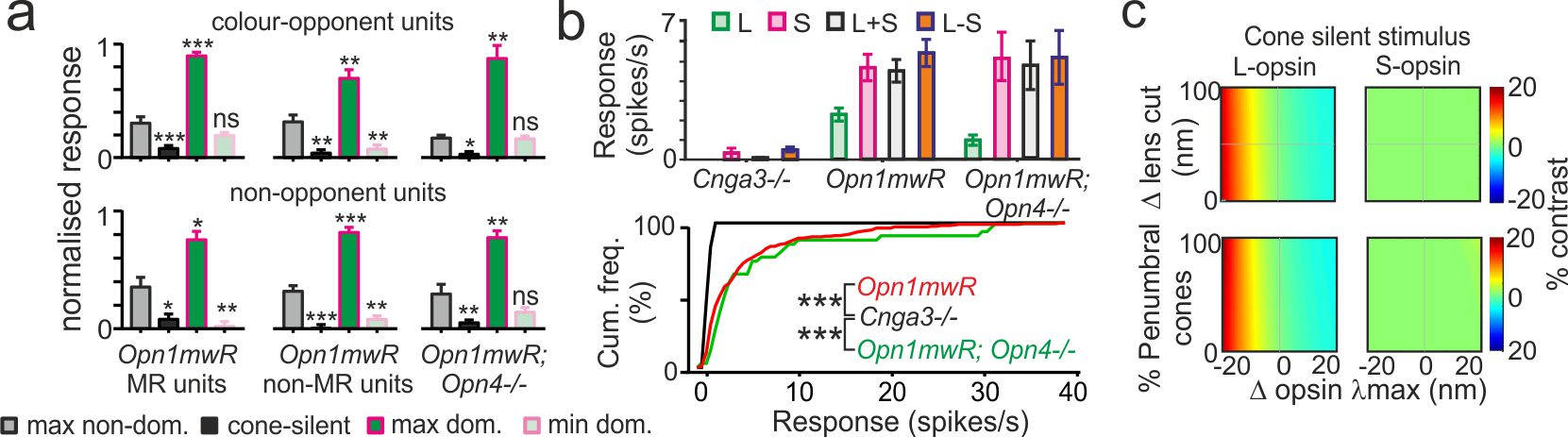

Supplement: Supplementary file 7 — Figure S7. Additional validation of cone-isolating stimuli. (a) Mean ± SEM responses of colour opponent and non-opponent MR and non-MR units in Opn1mwR and Opn1mwR;Opn4−/−mice that responded at ND1 (conventions as in Fig. 3b). Data were analysed by one-way RM ANOVA with Dunnett’s post-test (clockwise from top left n = 19, 18, 7, 17, 36 and 15). (c) Top: Mean ± SEM responses of Cnga3−/−, Opn1mwR and Opn1mwR;Opn4−/− neurons to 75% contrast cone-isolating stimuli; (analysis includes all light-responsive cells tested in all genotypes; n = 24, n = 230 and n = 41 respectively). Bottom: cumulative frequency distribution for maximal response evoked by cone-isolating stimuli in the same populations of cells. Data were analysed by Kruskal-Wallis test with Dunn’s test for multiple comparisons. *,**,*** indicate P < 0.05, P < 0.01 and P < 0.001 respectively; ns indicates P > 0.05. (c) Changes in cone-opsin contrast for cone-silent stimuli as a result of varying both λmax and contribution of penumbral cones (conventions as in Fig. 3f, i). (JPG 135 kb) [file 12915_2018_552_MOESM7_ESM.jpg]

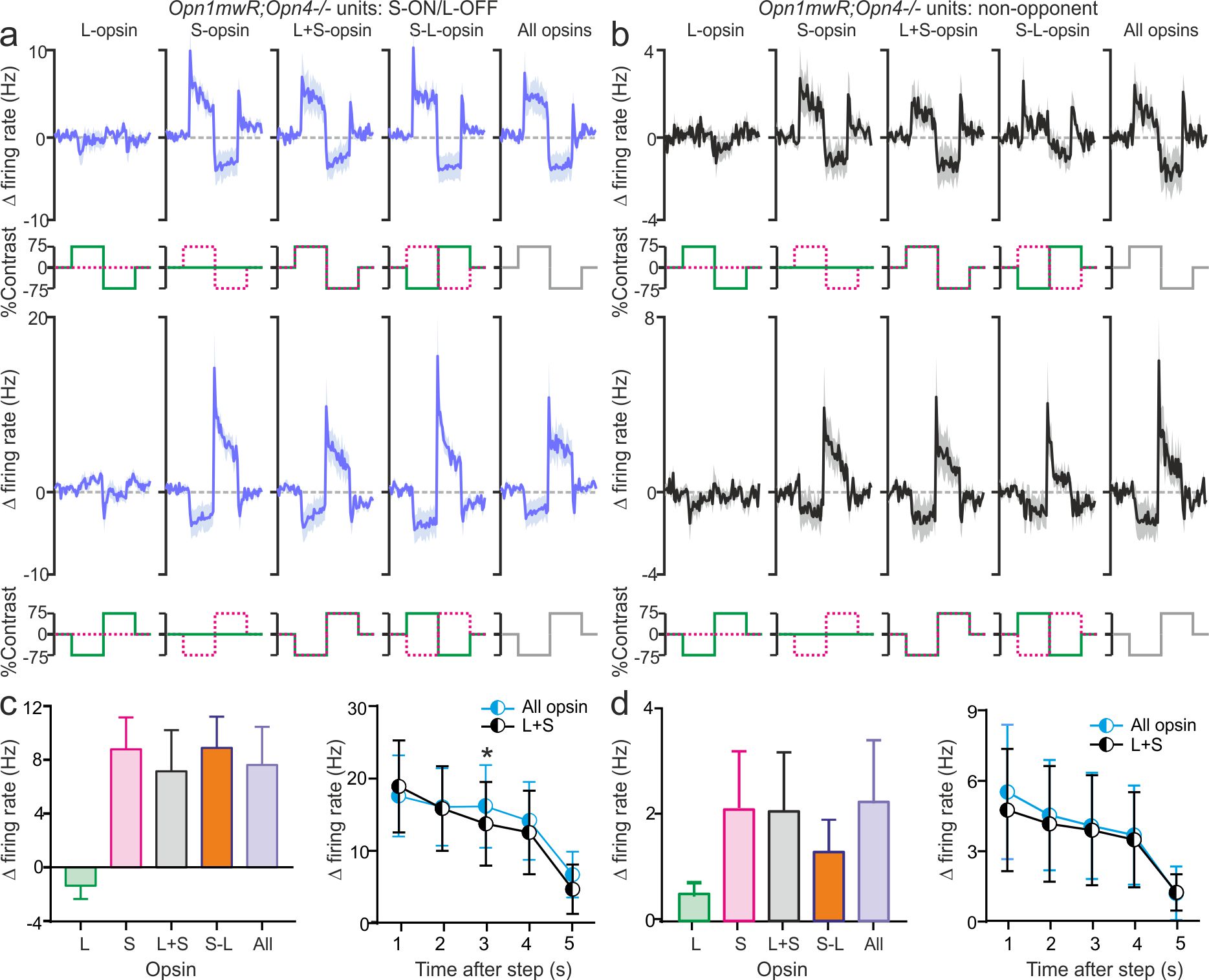

Supplement: Supplementary file 8 — Figure S8. Opponent and non-opponent responses of pretectal neurons from Opn1mwR;Opn4−/− mice under the stimulus paradigm used for pupillography. (a, b) Mean ± SEM baseline subtracted responses of S-ON/L-OFF (a; n = 6) and non-opponent (b; n = 18) Opn1mwR;Opn4−/− units (from 4 mice) to 75% contrast cone isolating and all opsin stimuli delivered as for pupillography (Fig. 5). (c, d) Left: Mean ± SEM change in firing between ‘dim’ and ‘bright’ stimulus phases for all stimuli (averaged across full 5 s phase and both stimulus polarities) for S-ON/L-OFF (c) and non-opponent units (d) as above. Right: Mean ± SEM change in firing between ‘dim’ and ‘bright’ stimulus phases for 75% contrast stimuli targeting L + S cone opsins or all photoreceptors as a function of time since contrast step (averaged across both stimulus polarities as above). Data analysed by two-way RM ANOVA with Sidak’s post-tests. Non-opponent units lacked any stimulus-related differences, but a nominally significant increase in S-ON/L-OFF neuronal responses at 3 s but not earlier or later timepoints was observed (P = 0.03). (JPG 294 kb) [file 12915_2018_552_MOESM8_ESM.jpg]

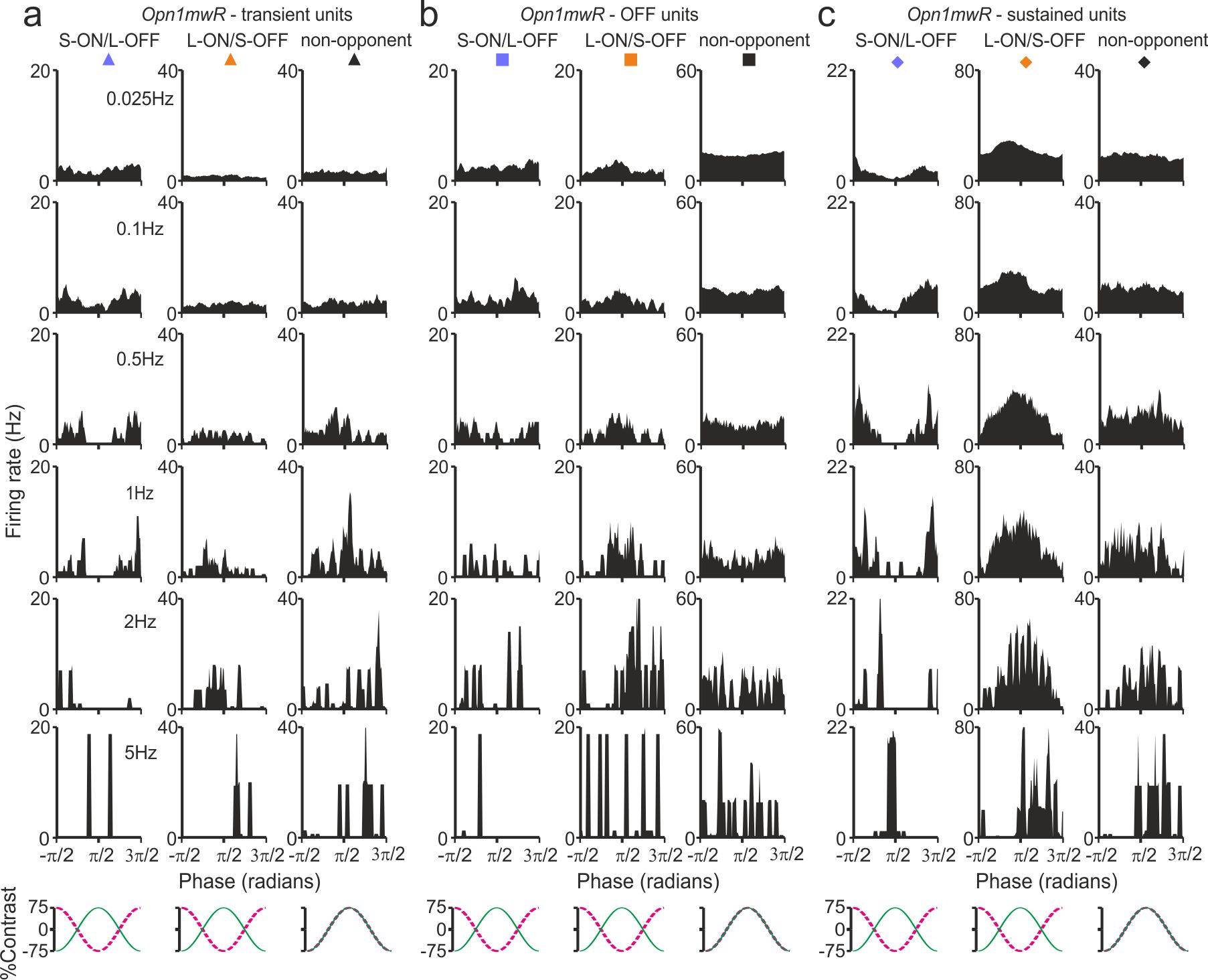

Supplement: Supplementary file 9 — Figure S9. Temporal tuning of cone inputs to non-melanopsin-responsive cells. (a-c) Example peristimulus firing rate histograms for transient (a), OFF (b) and sustained (c) non-MR neurons in Opn1mwR mice, tested with sinusoidal oscillations of their optimal cone stimulus type (L − S modulation for chromatic units and L + S stimulus for the non-opponent units – rightmost traces in each panel) at 75% contrast and varying temporal frequency. (JPG 275 kb) [file 12915_2018_552_MOESM9_ESM.jpg]

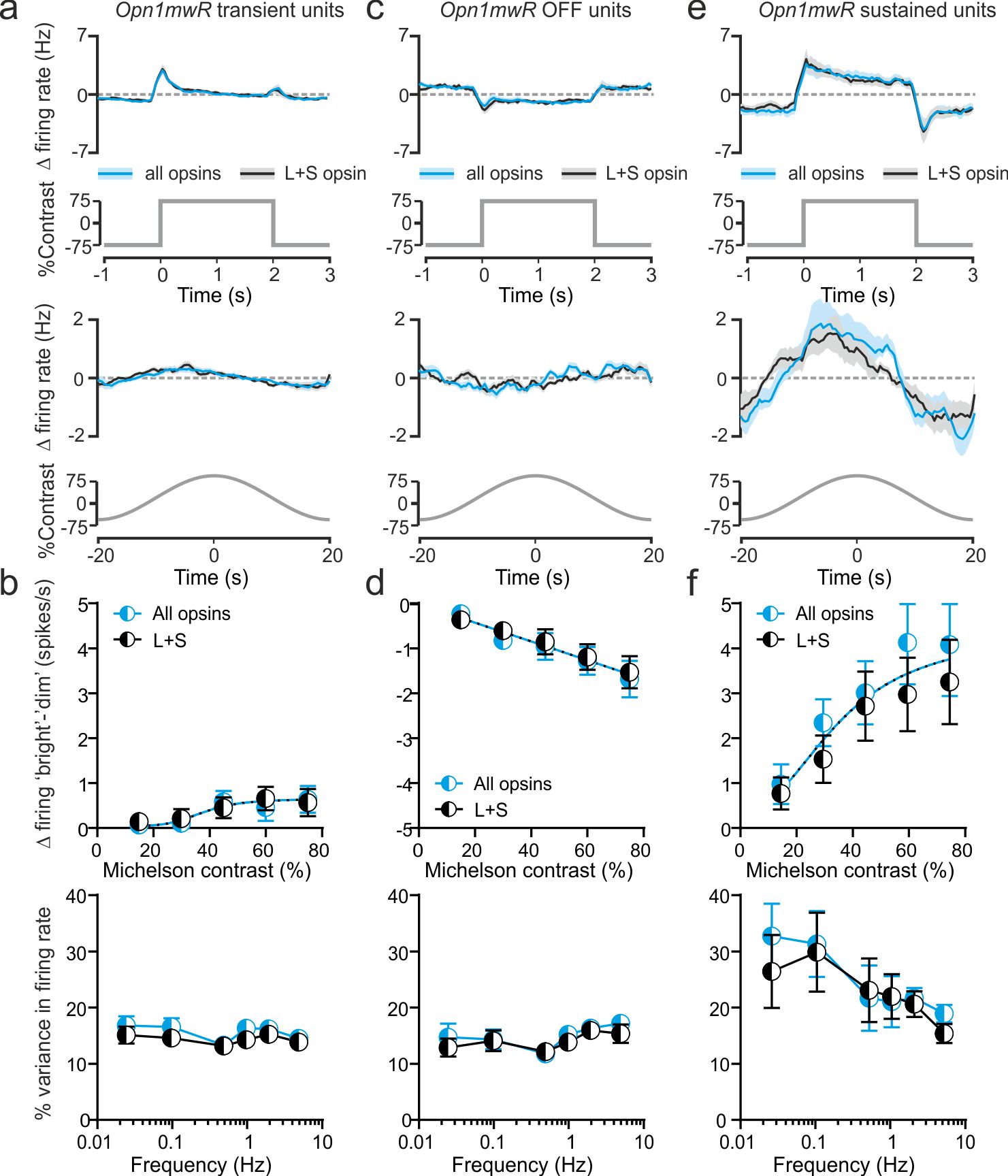

Supplement: Supplementary file 10 — Figure S10. Non-melanopsin-responsive neurons display equivalent responses to stimuli activating both cone opsins in the presence or absence of contrast for other photoreceptors. (a, c, e) Mean ± SEM responses of Opn1mwR transient units (a; n = 62) and OFF (c; n = 16) and sustained (e; n = 11) cells to rapid (0.25 Hz square wave; top) or gradual (0.025 Hz sinusoid; botom) spectrally neutral stimulus modulations (all opsins) and stimuli targeting just L- and S-cone opsin (75% contrast). (b, d, f) Contrast (top) and temporal frequency (bottom) tuning curves for Opn1mwR transient (b), OFF (d) and sustained (f) responses to all opsin and L + S-opsin-isolating stimuli (as above). For contrast response analysis, data points represent difference in mean firing rate during the last 400 ms at ‘bright’ vs. ‘dim’ stimulus phases. For temporal frequency analysis data points represent the % variance in firing rate accounted for the stimulus. In both cases data analysed by two-way RM ANOVA with Sidak’s post-tests. *** = P < 0.001. (JPG 269 kb) [file 12915_2018_552_MOESM10_ESM.jpg]

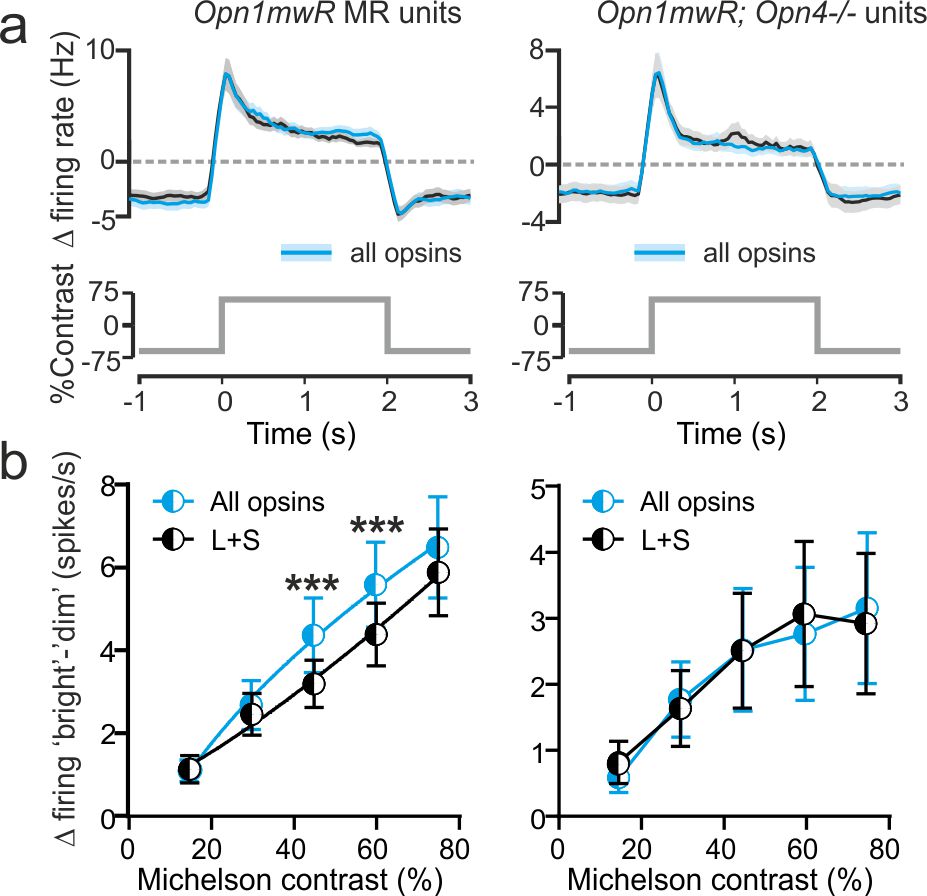

Supplement: Supplementary file 11 — Figure S11. Responses to cone-selective and all-opsin contrast at lower irradiance. (a) Mean ± SEM responses of Opn1mwR MR (left; n = 34) and Opn1mwR;Opn4−/− (right; n = 25) units tested at ND1 with 60% contrast stimuli modulating L + S opsin or all-opsins. (b) Contrast tuning curves for Opn1mwR MR (left) and Opn1mwR; Opn4−/− (right) responses to all opsin and L + S-opsin-isolating stimuli (as above). Data points represent difference in mean firing rate during the last 400 ms at ‘bright’ vs. ‘dim’ stimulus phases. Data analysed by two-way RM ANOVA with Sidak’s post-tests. *** = P < 0.001. (JPG 113 kb) [file 12915_2018_552_MOESM11_ESM.jpg]
